# Development of a toolkit to improve interprofessional collaboration and integration in primary care using qualitative interviews and co-design workshops

**DOI:** 10.3389/fpubh.2023.1140987

**Published:** 2023-04-17

**Authors:** Muhammed Mustafa Sirimsi, Hans De Loof, Kris Van den Broeck, Kristel De Vliegher, Paul Van Royen, Peter Pype, Kristel Driessens, Emily Verté, Roy Remmen, Peter Van Bogaert

**Affiliations:** ^1^Department of Family Medicine and Population Health (FAMPOP), Faculty of Medicine and Health Sciences, University of Antwerp, Antwerp, Belgium; ^2^Centre of Research and Innovations in Care, Faculty of Medicine and Health Sciences, University of Antwerp, Antwerp, Belgium; ^3^Laboratory of Physiopharmacology, Faculty of Pharmaceutical Sciences, University of Antwerp, Antwerp, Belgium; ^4^Department of Nursing, White-Yellow Cross of Flanders, Brussels, Belgium; ^5^Center for Family Medicine, Department of Public Health and Primary Care, Faculty of Medicine and Health Sciences, Ghent University, Ghent, Belgium; ^6^Department of Sociology, Faculty of Social Sciences, University of Antwerp, Antwerp, Belgium; ^7^Department of Family Medicine and Chronic Care, Faculty of Medicine and Pharmacy, Vrije Universiteit Brussel, Brussels, Belgium

**Keywords:** primary care, interprofessional collaboration, integrated care, sociocracy, psychological safety, public health, general practice

## Abstract

**Background:**

Despite numerous attempts to improve interprofessional collaboration and integration (IPCI) in primary care, patients, care providers, researchers, and governments are still looking for tools and guidance to do this more efficiently. To address these issues, we decided to develop a generic toolkit, based on sociocracy and psychological safety principles, to guide care providers in their collaboration within and outside their practice. Finally, we reasoned that, in order to obtain integrated primary care, different strategies should be combined.

**Methods:**

Development of the toolkit consisted of a multiyear co-development process. Data originating from 65 care providers, through 13 in-depth interviews and five focus groups were analysed and subsequently evaluated in eight co-design workshop sessions, organised with a total of 40 academics, lecturers, care providers and members of the Flemish patient association. Findings from the qualitative interviews and co-design workshops were gradually, and inductively adapted and transformed into the content for the IPCI toolkit.

**Results:**

Ten themes were identified: (i) awareness of the importance of interprofessional collaboration, (ii) the need for a self-assessment tool to measure team performance, (iii) preparing a team to use the toolkit, (iv) enhancing psychological safety, (v) developing and determining consultation techniques, (vi) shared decision making, (vii) developing workgroups to tackle specific (neighbourhood) problems, (viii) how to work patient-centred, (ix) how to integrate a new team member, and (x) getting ready to implement the IPCI toolkit. From these themes, we developed a generic toolkit, consisting of eight modules.

**Conclusion:**

In this paper, we describe the multiyear co-development process of a generic toolkit for the improvement of interprofessional collaboration. Inspired by a mix of interventions from in and outside healthcare, a modular open toolkit was produced that includes aspects of Sociocracy, concepts as psychological safety, a self-assessment tool and other modules concerned with meetings, decision-making, integrating new team members and population health. Upon implementation, evaluation and further development and improvement, this compounded intervention should have a beneficial effect on the complex problem of interprofessional collaboration in primary care.

## Introduction

The number of people with chronic conditions has increased relative to the total population, resulting in a greater need for primary care (PC) professionals to collaborate interprofessionally and strengthen relationships with one another (1, 2). Working in mono-and multidisciplinary group practices offers new possibilities and challenges in the context of care continuity and care coordination (3, 4). Kringos et al. (5) indicated that a strong primary care system with a patient-centred approach can provide answers to the current challenges care providers are facing. They presented the following four innovations to handle the challenges: encouragement of cooperation between care providers, providing new payment systems and incentives for integrated and community care, making cooperation and teamwork a high priority, and enhancing a patient-centred care approach. However, collaboration with different professionals around a patient is not always easy, and asks for important skills to overcome difficulties within teams (6, 7).

Over the past decades, several attempts have been made to define interprofessional collaboration and determine strategies to enhance cooperation between healthcare providers (7–10), using well-known strategies and methods that have been broadly described in the healthcare literature (11, 12). However, some industries (e.g. ICT and automotive industry), have been sustainably adopting other ideas and practices to improve collaboration and integration, such as psychological safety and Sociocracy (13–16). These might be reusable in healthcare settings (17–21). Edmondson et al. described psychological safety as a shared belief that the team is safe for interpersonal risk-taking. (20, 22–24) Furthermore, Newman et al. identified psychological safety as a critical factor in the understanding of voice, teamwork, team learning, and organizational learning (21). In a psychologically safe working environment, team members should feel comfortable, and unconcerned about being embarrassed, rejected, or punished for speaking up (21, 23, 25). By fostering an environment of greater psychological safety, organisations can maximize everyone’s skills and competencies (21, 25). That’s why many companies such as Google used this concept to bring up innovative ideas or facilitate product development (20). Although the concept of psychological safety offers many possibilities to achieve interprofessional collaboration and integrated care, it does not cover all aspects of interprofessional collaboration and integration. Additional concepts and practices are needed.

Sociocracy 3.0 (S3) is based on a governance model that focuses on the equality of individuals (26–30). It is built on seven principles that shape organizational culture: effectiveness, consent, empiricism, continuous improvement, equivalence, transparency, and accountability (30, 31). These principles are reflected in all facets of S3 and by understanding them, implementation of S3 is facilitated (30). In Sociocracy, decisions are made based on “consent.” This means that a decision can be made if there are no overriding objections from the team members against making that decision (30–32). If there are substantial objections, the proposal will be amended until the objections are resolved (26, 29). To avoid the trap of consensus, explicit consent to a decision by all team members is necessary. This means that, when making decisions, the range of tolerance of all team members will be taken into account, and final decisions should be located within this range of tolerance. If that is not possible, the proposal should be adapted in such a way that it fits the range of tolerance. In some democratic governance forms, a tyranny of the majority is a possibility, but in S3 all ideas get consideration (30–33). In S3, team meetings are exemplified with a circle composed of equal team members (30, 32–34). Communication in these circles happens in rounds enabling everyone’s chance to speak (30, 32). Each new round starts with a different person, and reverses the direction to add variation in the sequence of opinions (30, 32).

Though many isolated interventions and strategies have been used to improve interprofessional collaboration and integration in primary care (35–40), none of them have been shown to be sufficient to reach integrated care on its own (37). We reasoned that, in order to obtain integrated primary care with the existing materials, these strategies could be combined. Therefore, we decided to develop – in co-creation with a lot of professionals, patient representatives, and academics – a toolkit that adapts and adopts existing strategies and methods from in and outside healthcare. We aimed to develop a practical toolkit that could be used by all types of primary care workers and practices, containing single tools that could flexibly be used to encourage collaboration across settings and care providers of all kind. Building further on a scoping review inventorying effective strategies for integrated care, this paper describes the process of development of the toolkit in Flanders, the Dutch-speaking region in Belgium (41).

In this paper, we describe the process of development of the toolkit. This included inventorying (i) the strategies, methods and tools that are used in Flemish primary care teams to achieve efficient interprofessional collaboration and integration (IPCI) and desirable outcomes; (ii) strategies and methods from in and outside healthcare that could be adapted/adopted into the toolkit; (iii) and implementation and evaluation strategies of interprofessional collaboration and integration (IPCI) in primary care.

## Methodology

### Study design

The toolkit is based on data originating from primary care professionals, collected in several semi-structured interviews and co-design workshops, organised with professionals, academics and members of the Flemish patient association. In addition all interviews, interview guides co-design workshops and tools were performed or developed in Dutch.

#### Semi-structured interviews

We used a qualitative, inductive approach to explore the experiences of primary care professionals towards interprofessional collaboration and integration in primary care. In addition, we tended to identify appropriate strategies and methods, used by primary care professionals to facilitate or improve interprofessional collaboration and integration. The semi-structured interviews were performed by MMS (PhD student) in two stages, using three different interview guides. (see Supplementary material) This researcher was trained in qualitative research methods and performed previous qualitative research (42–44).

We applied the following inclusion criteria to select practices: (i) they were established in Flanders, the Dutch-speaking part of Belgium, (ii) they were multidisciplinary settings, including at least two different disciplines, and (iii) they were officially recognized by the Flemish Government as healthcare settings. We invited all team members who worked in the practices to participate in the focus groups and included professionals who worked full-time (or at least 80%) and had experienced the establishment of the practice in the interviews.

#### Co-design workshop sessions

In total eight co-design workshop sessions were performed throughout the whole development process. Due to Covid-19 measures, these workshops were held online and were recorded after obtaining the participants’ consent. Academics, practitioners, lecturers of different professional backgrounds and a member of a patient association participated actively in these workshops to co-develop the IPCI-toolkit (Table 1).

**Table 1:** Overview of co-design workshop characteristics.

Workshop characteristics	
# Sessions	8 workshop sessions
Average duration	90–150 min/session
# Organisations	Universities: 4 University colleges: 5 Patient association: 1 (Home) nursing organisation: 1
Total # participants	40 participants + presenter + moderators
Profiles	General practitioners (GP’s), nurses, physiotherapists, social workers, sociologists, psychologists, pharmacists, and dieticians
Occupation	Academics, lecturers, practitioners, and patient representative

All participants received an email beforehand with the questions asked during the workshop. Depending on the particular session some prototype elements of the toolkit necessary to prepare for the workshops were sent upfront.

Every session started with a presentation in which MMS presented the state of progress and the newest findings of the study. During this presentation, all participants were able to ask questions, and subsequently, KV, HL, KB, and PB in turn moderated the remaining parts of the workshop, each moderating a question. All participants answered the questions irrespective of their background in rounds as adapted from the S3 circle framework (30, 32). This was repeated until participants had no further comments (31).

### Sampling and participants

#### Interviews and focus groups

We used purposive random sampling strategy to include participants for our study, specifically maximum variation sampling. The potential participants were contacted through the PC practices where they worked. To initiate contact, we sent an email to each PC practice that outlined the purpose of the study, their role in it, and described our research project. Finally, we requested the PC practices to invite all eligible team members to participate in our study. No relationship was established prior to this study.

Data collection continued until we achieved data saturation, which is a point that collection of additional data no longer yields new insights. In addition, using a maximum variation sampling allowed us to ensure that we had collected a diverse set of perspectives and experiences from participants from different backgrounds and roles within their respective PC practices.

#### Co-design workshops

To select participants for the co-design workshops, we invited all members of the greater research team of working package five. Participants were contacted through email, which included information about the project and the specific workshop topic, as well as our expectations for their participation.

The selection was based on the involvement in the research team of working package five, expecting that their experience and expertise would provide valuable insights for the development of the toolkit. By including all team members we ensured that we captured a diverse range of perspectives and feedback from individuals with different roles, experiences, and backgrounds. Moreover, we were able to use the collective knowledge to co-design a toolkit to improve interprofessional collaboration and integration in primary care.

#### The two-year development process

**Figure 1:**
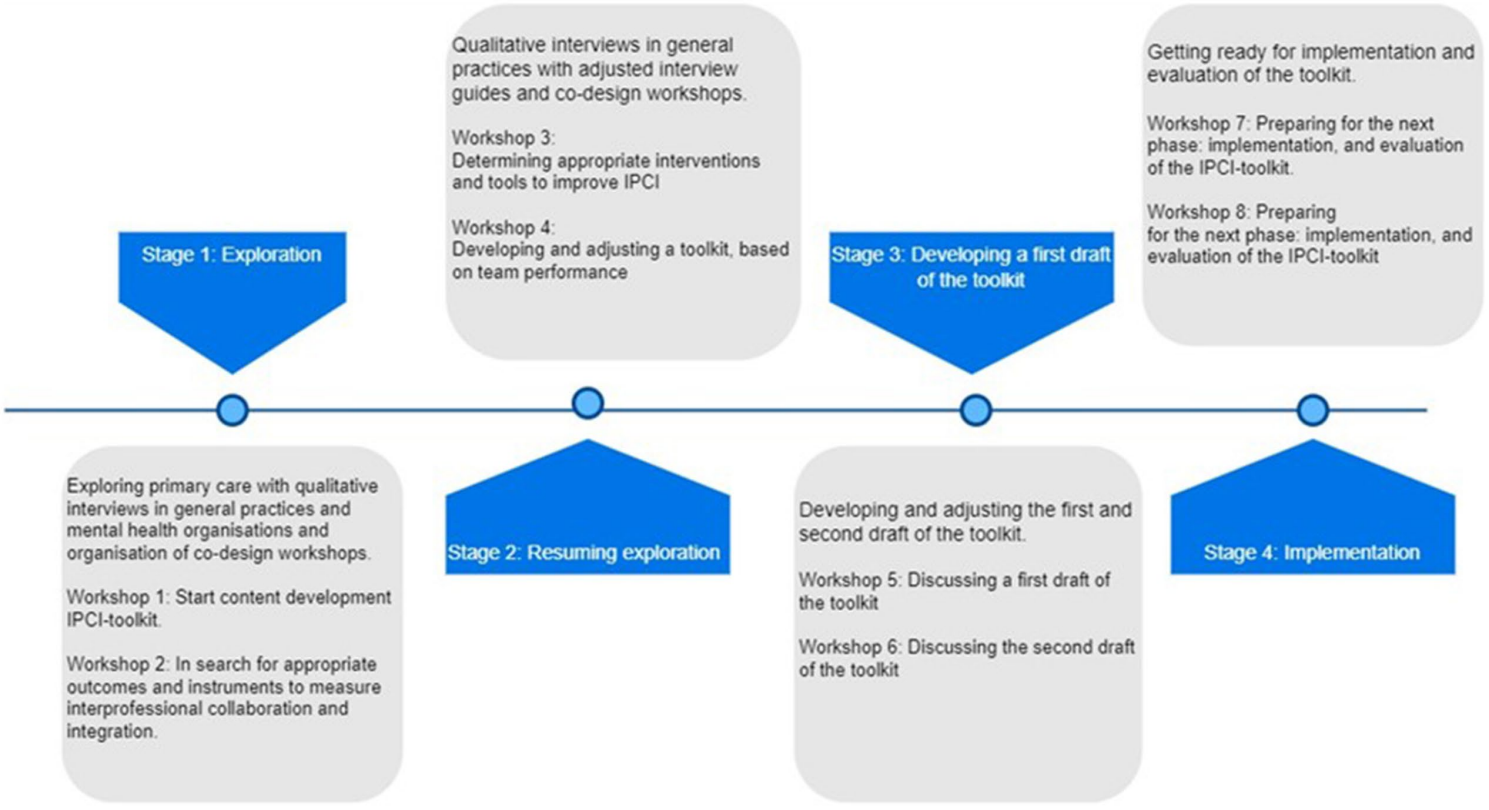
An overview of the two-year development process of the IPCI-toolkit* existing of four stages: exploration, resuming exploration, developing a first draft of the toolkit, and implementation. *IPCI, Interprofessional collaboration and integration in primary care.

The two-year toolkit development process consisted of (i) qualitative interviews with primary care professionals, (ii) co-design workshop sessions, (iii) content development, and (iv) IPCI-toolkit build-out. An overview of the development process is shown in Figure 1. Findings from the qualitative interviews and co-design workshops were gradually, and inductively adapted and transformed into the content for the IPCI-toolkit, using a sociocracy framework (26, 27, 30) (Figure 1). More specifically, MMS analysed the collected data and subsequently, these analyses and findings were reviewed and approved by the researchers HL, KB, KV, and PB independently. Microsoft Excel was used to manage the collected data. Finally, Consolidated criteria for Reporting Qualitative research Checklist was used to check our manuscript (45).

#### Stage 1 (January – September 2020): Qualitative interviews and co-design workshops

In stage 1, a total of 11 in-depth interviews and two focus groups were conducted in four general practices and four mental health care settings. The interviews were transcribed ad verbatim and the following topics were discussed through the semi-structured interview guide: (i) how healthcare providers experience the current collaboration, (ii) the organisation of team meetings, (iii) information-sharing in the team, and (iv) interventions and strategies to improve collaboration and integration in primary care. A detailed overview of the research characteristics regarding the interviews and focus groups in this stage is presented in Table 2.

**Table 2:** Overview of research characteristics in stage 1.

Setting	General practice	Mental health care
Interviews	5 in-depth interviews 1 focus group	6 in-depth interviews 1 group interview
Total duration interviews	321 min	412 min
Number of settings	4 practices	4 practices
#Participants	36 caregivers	10 caregivers
Education	GP’s: 6 Nurses: 28	Psychologists: 7 Psychologist + sexologist: 1 Applied psychologist: 2
Role in the team	/	Leaders: 4 Frontline professionals: 6

Based on the findings of the interviews and focus groups during stage 1, the first two co-design workshop sessions were organised (see Table 3 for the workshop set-up and participant characteristics). After performing discussions during the workshops, we decided to continue our data collection in multidisciplinary general practices. Besides, additional focus groups were needed to gain more insight into the teamwork and the team dynamic of general practices. For this reason, a new semi-structured interview guide (see Supplementary material) was developed for stage 2, to be deployed in multidisciplinary general practices only (Tables 2, 3).

**Table 3:** Overview of the co-design workshop sessions: topic, date, number of participants, background participants, and the questions.

Workshop 1	Topic: Content development IPCI-toolkit
	Date: 29 May 2020
	Participants: 6
	Background participants: Physiotherapists (*N* = 1), dietitians (*n* = 1), nurses (*n* = 2), nurse/gerontologists (*n* = 1), GP’s (*n* = 1), social workers: (*n* = 1)
	Questions: When is a team an added value for providing optimal care? Give 1 example of an intervention that affects team functioning? How can we ensure that the patient is included in this collaboration? What’s in it for the patient from this collaboration? How to inform the patient that he/she is being treated by a team?
	Expected outcomes: An estimate of the intervention needs to improve interprofessional collaboration and integration in primary care Strategies: to develop a toolkit, beneficial for the patient. To inform the patient about the collaboration and coordination of their care team
Workshop 2	Topic: Outcomes and instruments to measure IPCI
	Date: 5 June 2020
	Participants: 4
	Background participants: physiotherapists (*n* = 1), nurses (*n* = 1), GP’s (*n* = 1), members of the Flemish patient association (*n* = 1)
	Questions: Which aspects should we measure as a matter of priority within the model of the Quadruple Aim (patient, population, care provider and cost-efficiency) to gain insight into the degree of quality of interprofessional collaboration and integration and which instruments can we use for this? Which PROMs/PREMs are available to measure outcomes on health and well-being in primary care? Can these measurements be used to score the entire team? How can we measure interprofessional collaboration and integration from the patient’s point of view? And how does this relate to the self-assessment of a team? Which outcomes indicate the relationship/connection of interprofessional teams with the community/environment? Which instruments can we use to measure this? Which techniques prevent “gaming” of outcome measurements? Give 1 piece of advice. Gaming = (Sub)consciously choosing for one’s advantage at the expense of efficient and effective patient-oriented care.
	Expected outcomes: Appropriate outcomes and instruments to measure IPCI in primary care settings. Content for a toolkit, which can bring measurable changes to teamwork (IPCI) Strategies to transform, adapt, and adopt knowledge from international literature, which could be used as input for the toolkit. Strategies to maintain a patient-centred approach and avoid bias and gaming.

#### Stage 2 and 3 (September 2020 – November 2021): Semi-structured interviews in general practices and the outline of co-design workshops

In a second stage, we performed a qualitative study consisting of semi-structured interviews with care professionals working in general practices and subsequently four co-designing workshops. A total of two in-depth interviews and three focus groups were conducted in four general practices, with 19 caregivers. A semi-structured interview guide was used, addressing the following topics: (i) structure of the team, (ii) shared goals and vision, (iii) collaboration with team members, and (iv) coordination with and around the patient. The interviews were transcribed ad verbatim and thematic analysis was performed. A detailed overview of research characteristics is available in Table 4.

**Table 4:** Overview of research characteristics in stage 2.

Setting	General practice
# Interviews	2 in-depth interviews 3 focus groups
Total duration interviews	316 min
# Practices	4
# Caregivers	19 caregivers
Occupation	GP’s: 8 Nurses: 6 Physiotherapists: 1 Medical secretary: 1 GP trainee: 1 Psychologists: 1 Social workers: 1

Additionally, four co-designing workshops were organised in which the following topics were discussed: (i) determining appropriate interventions (WS3), (ii) developing and adjusting a toolkit, based on team performance (WS4), (iii) discussing a first draft of the toolkit (WS 5), and (iv) discussing the second draft of the toolkit (WS 6).

The focus of each co-designing workshop was as in Table 5.

**Table 5:** Overview of the co-design workshop sessions: topic, date, # participants, background participants, and the questions.

Workshops 3 and 4	Topic WS3: Determining appropriate interventions Topic WS4: Development and adjustment of the toolkit
	Date: 23 November 2020 and 26 November 2020
	Participants: 8 and 4
	Background participants workshop 3: member of the Flemish patient association (*n* = 1), GP’s (*n* = 2), physiotherapists (*n* = 1), nurses (*n* = 1), dietitians (*n* = 1), sociologists (*n* = 1), nurse/gerontologists (*n* = 1) Background participants workshop 4: GP’s (*n* = 1), Nurses (*n* = 2), social workers (*n* = 1)
	Questions workshop 3: What is your opinion on this statement? A measurement tool from the study, which characterizes team collaboration, can also help with a team “self-diagnosis” and the selection of possible interventions. Rank these interventions in importance Which interventions can be combined? Which combination of interventions yields synergies? Which interventions antagonize each other? What is your opinion about these statements regarding the implementation in Flanders based on interventions that we know from literature? We have to be very careful about this: we may be trying to solve problems that do not exist here! Just do it, despite implementation issues: because this puts the finger on the wound and is, therefore, part of the problem to be solved.
	Expected outcomes workshops 3 and 4 Determining appropriate interventions and tools to improve IPCI in primary care Developing content: Identifying influential factors of IPCI, according to primary care professionals. Determining which interventions or tools are (not) compatible, and synergic when used together. Developing content: Identifying strategies to transform, adapt, and adopt knowledge from international literature. Development of a self-assessment tool
Workshop 5	Topic: Discussing a first draft of the toolkit
	Date: 21 January 2021
	Participants: A total of 4
	Background participants: Nurses (*n* = 2), pharmacists (*n* = 1), psychologists (*n* = 1).
	Questions: When can we call this toolkit a success? Can we use short recordings/videos to introduce the modules in the toolkit? How can we facilitate the implementation of the toolkit? What if the practices are already implementing a toolkit or intervention? What does this mean for our interventions? How should the toolkit be structured to generate sufficient data? How can we structurally monitor the participating teams? What difficulties/pitfalls does the introduction of a dashboard entail? How do we deal with non-participation or stagnation of the process? Is a backup plan necessary?
	Expected outcomes: Discussing whether the first draft of the toolkit is suitable to improve IPCI in primary care. Identifying strategies to implement the toolkit in a longitudinal study. Identification, prediction, and preparation for potential problems, and obstacles in the implementation process.
Workshop 6	Topic: Discussing the second draft of the toolkit
	Date: 19/08/2021
	Participants: 5
	Background participants: Policy makers (*n* = 1), GP’s (*n* = 1), dietitians (*n* = 1), social workers (*n* = 1), nurses (*n* = 1)
	Questions: Are these tools suitable to strengthen interprofessional collaboration and integration in primary care? (Which are/not?) How can we facilitate the implementation of this toolkit? How should the toolkit be structured to generate sufficient data? Are the measuring instruments determined for the self-evaluation suitable? Expected outcomes: Discussing whether the second draft of the toolkit is suitable to improve IPCI in primary care. Identifying strategies to implement the toolkit in a longitudinal study. Strategies to upgrade the toolkit, based on data from the self-assessment tool.

Based on the data of the interviews and co-designing workshop sessions three and four, we developed a first and second draft of the toolkit (stage 3). This first draft was discussed and evaluated in the fifth workshop session. Based on the findings of the fifth workshop session, a second draft of the toolkit was developed. Subsequently, we organised a sixth co-designing workshop to discuss the second draft of the IPCI-toolkit and based on these findings we developed the last version of the IPCI-toolkit. (see Supplemental material).

#### Stage 4 (November 2021 – April 2022): Getting ready for implementation and evaluation of the toolkit

In a third stage, we performed two co-designing workshops to prepare the IPCI-toolkit for implementation in primary care settings and to identify strategies to evaluate teamwork and the impact of the IPCI-toolkit. We discussed: (i) the definition of efficient teamwork, (ii) adopting data from the self-assessment tool into the toolkit, (iii) strategies to facilitate the continuity of the implementation, (iv) how to deal with changing teams, and (v) foreseeing and anticipating the possible barriers while implementing (Table 6).

**Table 6:** Overview of the co-design workshop sessions: topic, date, # participants, background participants, and the questions.

Workshops 7 and 8	Topic: Preparing for the next stage: implementation, and evaluation of the IPCI-toolkit
	Dates: 11/10/2021 and 18/10/2021
	Participants workshop 7: *n* = 6 participants Participants workshop 8: *n* = 3 participants
	Background participants workshop 7: GP’s (*n* = 1), policy makers (*n* = 1), dietitians (*n* = 1), sociologists (*n* = 1), nurses (*n* = 2) Background participants workshop 8: GP’s (*n* = 2), Social workers (*n* = 1)
	Questions: How do you define a team and what determines whether a team is well attuned to each other? How can we design the pilot toolkit based on data from the self-assessment tool? How can we motivate healthcare providers to start with the toolkit and which steps can be taken to maintain this motivation and prevent the drop-out of practices or teams? How do we deal with fluctuating or changing teams? What can go wrong during the implementation of the toolkit? What about intellectual property? And what steps should we take to valorise the toolkit?
	Expected outcomes: Definition of “good” teamwork or a good collaboration. Definition of well-matched team members. Strategies to upgrade the toolkit, based on data from the self-assessment tool Identification, prediction, and preparation for potential problems, and obstacles in the implementation process.

## Results

We first elaborate on the results from the interviews, focus groups, and co-design workshops. From the qualitative data, the following 10 themes were identified: (i) the importance of interprofessional collaboration, (ii) the need for a self-assessment tool to measure team performance, (iii) preparing a team to use the toolkit, (iv) enhancing psychological safety, (v) developing and determining consultation techniques, (vi) shared decision making, (vii) developing workgroups to tackle specific (local) problems, (viii) how to work patient-centred, (ix) how to integrate a new team member, and (x) getting ready to implement the IPCI toolkit. These teams were underlying the construction of the toolkit. See Table 7 for more details on these themes. Next, we will outline the toolkit we developed.

**Table 7:** An overview of the themes and subthemes extracted from the development process.

Themes	Subthemes
Theme 1: Importance of interprofessional collaboration	N/A
Theme 2: The need for a self-assessment tool to measure team performance	N/A
Theme 3: Preparing a team to use the toolkit	Developing a shared vision Developing shared goals
Theme 4: Enhancing psychological safety	Achieving a lateral hierarchic structure Having trust in each others’ competencies and skills Having an open-culture
Theme 5: Developing and determining consultation techniques	Organising team meetings Informal team meetings Formal team meetings Digital meetings Building networks between care providers originating from different settings Coordination and role distribution
Theme 6: Shared decision-making	Achieving consensus and resolution of conflicts Documenting agreements
Theme 7: Developing workgroups to tackle specific (local) problems	N/A
Theme 8: How to work patient-centred?	N/A
Theme 9: How to integrate a new team member?	Recruiting a new team member Approaching a new team member
Theme 10: Getting ready to implement the IPCI toolkit	A mix of interventions Theory to practice Enabling the implementation of the toolkit

### Theme 1: Importance of interprofessional collaboration

According to care providers, a well-performing interprofessional collaboration can improve the quality of care. The multiple perspectives of different disciplines were reported as one important advantage. Multidisciplinary teams also provided better monitoring resulting in improved identification of complications.

*“For me, interprofessional collaboration is related with the care you give to a patient. Of course, if I worked alone as a general practitioner, I would never be able to offer the quality that we offer here as a team. […] I'm convinced of that!” “… For example, diabetes … The cooperation that we have in this regard ensures that the patient is often better monitored than that you as a doctor or nurse would do alone.” (GP)*

Next to strictly biomedical disciplines, it was important to have social and mental health workers in the practice.

*“… we have a social worker in our team, as well as a psychologist. A patient is not just a body. Just because everything is okay with the body doesn't mean the patient is okay. And I think since we now have those specializations in-house that we can expand (care providing)…” (GP)*

### Theme 2: The need for a self-assessment tool to measure team performance

Care providers from the interviews and focus groups indicated the willingness to measure their level of teamwork, but also reported the lack of accessible, deployable tools or information on current collaborative practices.

Participants of the co-design workshops, on the other hand, specified that there are ways to measure teamwork, but few were appropriate for use in primary care. During these workshops, the need for validated scales was raised and several existing scales and literature were presented. Workshop participants also recommended to measure more than only interprofessional collaboration by including aspects such as psychological safety and bio-psychosocial working. In addition, they proposed to also measure health conditions, working conditions, and job satisfaction of the care providers.

### Theme 3: Preparing a team to use the toolkit

#### Developing a shared vision

According to care providers, having a shared vision is an important requirement to practice and improve IPCI in primary care settings.

Participants distinguished long term and short term visions and listed their policy plans periodically. Nurses, GP’s, and other allied healthcare professionals indicated that developing this shared vision needs to happen in collaboration with all team members. Appropriate questions should be asked in meetings to reflect on the needs and preferences of the practices. The practices perform evaluations to assess the suitability of their vision regularly and checked whether modifications were necessary. If needed, their shared vision can be upgraded depending on contextual factors.

While developing a shared vision, it is important to maintain a patient and population centred approach. Care providers set their vision and goals to provide accessible care and included the wish of patients to become autonomous in their vision and goals. Additionally, some practices explicitly include concepts such as accessibility of care and patient-centred care in their shared vision.

*We all want to provide accessible care, we all want those patients to have low-threshold access to care. Above all, we want them to be as self-reliant as possible. This is the vision of that house for care and well-being (practice), that's what we stand for. (GP)*

*“What do we want to strive for as a practice?” Forming a vision for the team that every employee supports and is jointly responsible for. That's crucial.” (Nurse)*

#### Developing shared goals

When care providers with different backgrounds collaborate in an interprofessional team, developing common or shared goals are important. Most participating practices were successful in developing a shared vision, and they indicated that having realistic goals was also necessary to deliver “good care.” Practices distinguished year goals and end goals, and to reach these goals, having a coordinator in their setting was seen as facilitating. Hiring a coordinator reduced the other professionals’ workload and helped them to focus on their core duties, instead of spending time on administrative and managing tasks. This was experienced as “pleasant,” and they were convinced that, even if they could not hire a coordinator, they still needed a team member taking a coordinating role to facilitate the collaboration.

*Yes, we do indeed work with people and (work) patient-centred. But when it comes to collaboration, I think it doesn't matter if we work in a community health centre or another company. There is a need for […] someone who keeps the overview and who can see that, okay, ‘the company' needs this to be able to continue working or to be able to grow." (GP)*

### Theme 4: Enhancing psychological safety

#### Achieving a lateral hierarchic structure

According to care providers, a psychologically safe work environment starts with treating every team member equally and recognising their skills and competencies. Teams should not be allowed to maintain a hierarchy between nurses and doctors. In addition, care providers indicated that not only care providers but all personnel should be counted as equal team members.

*“I think everyone has trust in the other care providers and that you can therefore communicate openly. And that you shouldn't be afraid to say something. […] I do have the feeling that you can say your opinion here and that you are respected for it. If someone has a different opinion, it can just as well be broadening for yourself. To create a broader picture (perspective).” (nurse)*

Care providers indicated that having a lateral hierarchic structure was important to obtain an open culture, which means that all team members feel comfortable and can speak up for themselves. This lateral hierarchic structure increased the approachability/accessibility to ask for advice from colleagues, though achieving this lateral hierarchic structure required major adjustments from care providers with higher education or profile. Being “open-minded” and having trust towards lower educated colleagues seemed essential to facilitate teamwork.

*“That's also because we work quite horizontally here. And got the hierarchy out of it. So that also means that you go much faster […] as a nurse to a doctor to discuss something about a patient.” (nurse)*

*“You also have to be sufficiently open-minded, especially as a doctor. Because classically, of course, the doctor is above the others or has the final word. So as a doctor you have to be open-minded, [ … ] to try that. That might end well or I'll see how it goes. And afterwards I think the next step is trust, because you […] as a doctor, you're going to check everything about your colleague because you don't trust it. Therefore you cannot start by saying that we will […] work interdisciplinary.” (GP)*

#### Having trust in each others’ competencies and skills

Having trust in the capacity of a colleague enhances open communication, respondents said. Care providers should be able to trust and know that everyone is working as a professional, but at the same time care providers should understand that their colleagues are still human. A care provider cannot do everything and can make mistakes. Being able to talk about their mistakes and being able to rely on each other’s understanding is found to be important to deliver “good care.”

In addition, they indicated that the lack of mutual trust or respect between care providers could hinder the implementation of efficient care. Well-cooperating disciplines and the provision of quality care to chronically ill patients were thought to be interrelated.

*“Good cooperation between GPs and nurses is crucial. This is only possible through openness and through trust in everyone's abilities. This means that we believe that the skills and view of the nurses towards the patients is an added value for us as GP’s.” (GP)*

Good and open communication was found crucial for the collaboration between the different disciplines. Care providers wanted to feel comfortable and supported by their colleagues. This feeling increased their confidence and improved the collaboration between the GP’s and allied healthcare workers, which positively affected care continuity. In some cases, it was indicated that if acknowledgement and recognition for professionals’ skills and competencies were lacking, negatively influenced teamwork. Finally, organising team building activities regularly, and having fun together was presented as a facilitator for teamwork.

*“A GP trainee asked me if I knew how to perform an intramuscular injection. Here (in this case) she underestimated my knowledge and skills and therefore she found it difficult to trust the care of the patients to me.” (Nurse)*

*“Our team building, […] yes that is very classic of course. But that it can also promote group formation. And I also think it's important that you regularly have some fun with each other. […] That is the salve on the wounds that may be left by working together. (Nurse – coordinator)*

#### Having an open-culture

Care providers experienced “having an open culture” in a setting as very important for teamwork. The possibility to speak to anyone in one’s setting was encouraged and promoted, provided that their colleagues were also open to receive feedback. Care providers should have the opportunity to express themselves safely.

*“I think the worst thing that can happen, not just for triage, but also for other projects, is that there are frustrations that go unspoken. That's not good of course. When we say that something is not going well or that it is more difficult, this is certainly taken into account. Afterwards we will reconsider how we can approach this differently.” (GP)*

### Theme 5: Developing and determining consultation techniques

#### Organising structured team meetings

Care providers preferred structured team meetings. They searched for strategies to use these meetings optimally in terms of efficiency and effectiveness. This meant that they wanted to be able to organise team meetings without feeling that they are losing time, and they think that an external person (someone who is not a team member) may be very useful in this regard.

*“What if those team meetings were a little more structured?” Answer: “… it would increase efficiency enormously and yes, the things we have now tried to mix (to have a structured meeting) … that we no longer have to do that and that we can simply bring our expertise into it in terms of content. So I think it is very useful if you also have someone external for that. (an external person to develop structured meeting protocols)” (GP)*

The interviews showed that practices performed daily, weekly and in some cases monthly team meetings. During our study, we identified formal and informal team meetings. While both types of team meetings were used in the practices and provided an added value to teamwork, according to care providers, face-to-face contact seemed to be the most important and preferred communication technique.

*“Every 24 hours, around noon, we meet to discuss the past day. This meeting gives a lot of value every day, to see very quickly; 'What is wrong here and where are the bottlenecks?” (GP)*

#### Informal team meetings

Caregivers gave special attention to informal, face-to-face communication between team members. It was described as something which grows over time and becomes a culture in the practice. Every practice developed its habits and activities regarding informal communication. An example of this activity was lunching together. Moreover, most informal meetings happened while lunching.

*“The fact that we all eat together in the afternoon … in which work-related things are often discussed (in the meetings). So it is actually a regular habit that everyone eats together.” (Social worker)*

According to care providers, a more open culture was achieved because of these informal meetings. The barrier to meet each other, or to say “something” urgent whilst performing care lowered. This was mentioned as “being more accessible” towards each other. They indicated that, when lunching jointly, they were able to switch to a formal topic, if urgent cases occurred.

#### Formal team meetings

Care providers mentioned different types of formal team meetings. First, there were medical meetings, in which medical professionals were included (e.g. GP’s and nurses). In these meetings, care providers predominantly discussed cases of patients, which needed an interprofessional treatment. Secondly, they organised team meetings to discuss organisational matters. In these meetings, all team members were invited, and more practical matters of teamwork were discussed.

*“Yes there are weekly meetings. On Monday there is [ … ] a medical meeting. In which the doctors, nurses and very occasionally I participate as a physiotherapist. This mainly concerns substantive cases. On Thursday there is a kind of team meeting, [ … ] during which an update is mainly given by our coordinator. […] But at that moment cases are also discussed that are somewhat more complex, where that […] the paramedical branches also contribute.” (Physiotherapist)*

For these meetings, a logbook was drawn up and shared before the start of the meeting. This logbook consisted of the meeting topics, expectations, duration, and the necessary preparations. These meetings were experienced as an important activity to improve interprofessional collaboration and were recommended by all care providers. In general, they thought that the medical meetings could be more structured, and by using the right tools, they could be more beneficial.

*“That is actually based on how we perform meetings. It can be a bit chaotic at times. We do try to think outside the box. […] And I think there are tools for that, to make it a little more structured.” (GP-everyone agreed)*

Interprofessional team meetings had many advantages. Care providers indicated that by including multiple professions and disciplines in the team meetings, cases were discussed more thoroughly and they were able to find better solutions for their patients. In comparison to thinking and acting on their own, they were able to act upon and look at info from various perspectives. In addition, not using the available skills, competencies and experiences was seen as a waste of time and financial resources.

*“You can share a lot, you can bring all that expertise together in one point and everyone can work on it from their own point of view … Yes … we can think together about a client and that is just very useful because if you work on a certain line for a long time, the first, the second or the third line (primary, secondary, and tertiary care), then you only see your own line and it is good to be able to consult others and then coordinate … so I think that the fact we can think about it together is very, very useful.” (psychologist)*

#### Digital meetings

Another form of communication was digital communication, in which care providers made use of chatting applications, video meetings and electronic patient records. It was experienced as an easy way of communicating with each other. Especially the communication between primary care and secondary care settings proceeded mainly through this channel. Not working under one roof was less of an issue, and digital communication tools increased the accessibility of some care providers towards other settings and disciplines. Care providers also indicated that there is still a lot of work to be done to perform efficient digital meetings. Yet, developing integrated medical record files was seen as a responsibility of the government. Keeping those files up to date was mentioned as a major issue and an important barrier to performing “good care.”

*“I, the doctors and some nurses use an application. That is a kind of (medical) chat app […]. And I then send communication or specific medical data about a patient to other fellow physiotherapists. We also use that app regularly.” (physiotherapist)*

Despite the many advantages of digital communication, care providers opted that physical appearance was still needed to maintain a good collaboration. They preferred to see their colleagues in real life. Care providers indicated that there is a lack of interaction, and non-verbal expressions when meeting online. For this reason, they performed physical meetings as much as possible.

*“The idea was to do online meetings. We have advocated for that to continue live meeting. For myself, I find that meeting more convenient, when you see the people in person. […] I think you miss a lot in terms of interaction, in terms of expression. The little things you can pick up in person. And you have that much less with [online] meetings.” (Social worker – everyone in the focus group agreed)*

#### Building networks between caregivers originating from different settings

Interprofessional team meetings also took place with care providers from outside the practice. If needed, multidisciplinary consultations (MDOs) were organized for patients with complex health needs. In Belgium, these MDO meetings are financed by the government and are meant to discuss the situation of patients’ complex care needs multidisciplinary. According to care providers, it worked beneficial for mapping the care network of the patient. By meeting care providers from different settings regularly, the threshold for contacting care providers from different practices was lowered.

*“So regarding a MDO (multidisciplinary meeting in Flanders), you do hear from the patient who is involved, but they do not know the first and last name, for example. […] But that makes it difficult for us to know exactly who that is. There is also always a limit to calling or emailing someone you've never seen before. […] And after such an MDO it is easier, because you then have each other's e-mail addresses. You've seen each other before. So it is easier to contact each other afterwards.” (GP)*

According to care providers, being autonomous and self-sustaining made the construction of networks with external settings and organisations more accessible. Although team members were given more autonomy, they still required someone with a coordinating role to link the different organisations or settings. Teams preferred to have a connecting person between different settings. As this connecting person was lacking, many settings stopped collaborating.

*“It is necessary that every organization must have a connection, otherwise they will drop out … That is a very big advantage of (Mental health organisation), the faster you can be there, the more open people can go there, the faster the problem is solved … they do trust it and they also believe in the preventive function of (mental health organisation)” (psychologist)*

These connections and networks built by care providers seemed to facilitate information sharing about their patients. They indicated that they received and sent more referrals to these organisations after knowing more about each others’ work settings. As patients received care from the right care providers, the providers felt more comfortable treating patient profiles in line with their knowledge. Each practice or setting chose a common contact person to connect their settings. These common contact persons connected general practices with nursing, and mental health and welfare organisations, and brought innovations to the practices. Moreover, they were able to learn from these practices and care providers with different scopes.

*“You learn a lot about the other organizations. Even if you're not in it. And you get so much information. Every employee brings in an enormous amount of information into (the mental health organisation) And that is very useful. But also vice versa, they also take what they learn from each other back to their own teams. They already know about that in these teams, … (citing her colleagues) oh, be careful because within (another mental health organisation) they work differently or they do this or that.” (social worker)*

The practices differed from each other and had specific needs which distinguished them. However, these differences in needs did not bring any disadvantages to their collaboration. The different structure of every organization or setting was acknowledged when building networks and this was seen as an added value.

*“You notice the differences between the organizations when you hear more background. But that does not affect our way of working together. It's not like there are downsides or anything like that. You get to know the organizations a little more from the inside through your colleagues.” (GP)*

#### Coordination and role distribution

Coordination and the distribution of roles and responsibilities were important to achieve efficient teamwork. Care providers wanted to be able to share their responsibilities and tasks, without losing their freedom or feeling hindered by their colleagues. According to care providers, clear role distributions and responsibilities were needed, so that every caregiver was able to understand and perform according to their duties. Uncertainty about the division of roles led to mutual irritations, conflicts and inefficient patient care.

*“I have the idea that our coordinator mainly creates the setting in which we can work, so that it actually offers a lot of structure. So the substantive work, that we have a lot of freedom in that. Yes, that he really holds and creates a framework in which we can do our work … So yes, she guides us … So I think that's very good … But I never get the impression that she really controls how we design it.“(GP and everyone agreed)*

*“This can be done through consulting regularly, but also by agreeing on very clear things. So that everyone knows, that's my job, that's not my job. There must also be a continuous dialogue about this.” (nurse)*

Allied caregivers were assigned additional, advanced care roles next to their core duties, such as drawing up care trajectories and conducting one on one consultations with patients. As a result, some roles of the GPs and allied caregivers had similarities and even overlapped in certain situations, which was experienced as time-saving, and increased interdependency between care providers.

*“My main task is the care of chronically ill patients with COPD, diabetes, etc. In addition, I support the GP at busy times with the removal of stitches and blood tests.” (nurse)*

This was also the case with mental health and social workers.

*“I was actually hired as a social case manager. I support people with psychosocial problems who need extra help, for example through intensive home visits. This gives the doctor more time to help other patients.” (nurse – social case manager)*

Concrete agreements and written protocols were necessary to determine the quality of care. These protocols were drawn up by both GP’s and allied caregivers and facilitated the performance of team meetings, medical interventions, and the organisation of the practices. According to care providers, an evaluation from an external consultant, who specialised in management and organisation, was beneficial to the development of protocols, and to guiding all team members equally. This external consultant engaged in order to evaluate the protocols provided feedback, and suggested adjustments if needed. Care providers were able to fall back on these protocols if they deviated from standard care in complex patient cases.

*“The nurses also draw up protocols for the practice. They have the necessary knowledge and skills from their training for this. An outside physician evaluates and rewrites protocols for our practice four hours a week. This ranges from drafting a household e-mail to the injection technique of an insulin pen for a diabetic patient. This will then be sent back to us by email. Once you are up to date with the protocols, you are no longer dependent on colleagues …” (GP)*

### Theme 6: Shared decision making

#### Achieving consensus and resolution of conflicts

To facilitate decision-making processes between professionals, care providers developed decision-making protocols. These protocols were, in the first stage, developed with a selection of care providers depending on their profile, availability and motivation. After finishing the first draft, the protocol was presented to the remaining team members in a meeting. This way, every team member was in some way involved in every decision-making process and was able to give feedback or request adjustments if needed.

*“About the decision making in your practice. How do you make decisions? (question) Answer: We have rolled out a plan with that working group, showing how we are going to approach this. That is a decision that we have made as a working group, but that is then fed back at a team meeting. Look, this is the plan we have, do you agree? So, it is not the case that you are involved in every decision, but an agreement is always requested and feedback is also given.” (GP)*

*“I think it is important that everyone can speak freely. I think it is very important in such practices that everyone's expertise is actually recognised (validated) …” (nurse)*

According to care providers, in a decision-making process, it was important that every team member was able to speak up, and that everyone had the feeling that their expertise and input were respected equally. Having shared responsibilities and performing brainstorming about certain issues as a team was experienced positively by the care providers. On the other hand, they indicated that they wanted to retain a certain form of autonomy to make their own decisions.

*“You actually have the opportunity to […] closely monitor the patient and make certain decisions about their care yourself. […] You work together and make decisions together. But you can also make independent decisions and I think that is really an added value.” (nurse)*

To facilitate decision-making processes, some care providers were advised to follow training. These care providers were given pieces of training to improve their communication skills, which had several advantages in conflict resolution.

*“What did surprise me in my early days was that a lot of effort was put into communication skills. I can name several courses that I have followed here, about communication with colleagues, connecting communication. And at the moment you think, okay, this is something I can do now, can I use it or not. But when conflicts occur, it turns out to be useful that you followed that.” (nurse)*

#### Documenting agreements

When an agreement was reached after a decision-making process, care providers documented these agreements in a clear way and shared the documents with all participants, including colleagues who could not participate in the meeting. This way, they were able to refer to their agreements when necessary and evaluate their achievements in the long term.

*“I think it comes down to making very good agreements. We come from different organizations but we are also colleagues and just human beings. And yes, at the level of our small cooperation, I think that's the most important. And this team is doing well so far.” (GP)*

### Theme 7: Developing workgroups to tackle specific (local) problems

In some practices, workshops were developed to solve problems in and around the settings. These care providers distinguished between workgroups directed towards care providers and workgroups directed towards their patients. They used these workgroups to develop medical policy plans or to solve problems in a patient-centred way.

*“… so the working groups that we have. These are based on what we think is needed for the practice. For example, (to develop) a medical policy plan. Now, we developed a new medical policy plan. And are waiting for a working group for sexual health and a working group for Advanced Care Planning (ACP). Because we notice that, for example, for unwanted pregnancies … in the practice, that contraception that that's not going well. And we have a low percentage of women who come in to give pap tests, so there's a need for that. So then you start a working group.” (GP)*

According to care providers, they were selective when choosing participants for the workgroups. They chose care providers who had the appropriate profile and background to solve the specific problems. Besides, they looked for motivated team members, who were interested in the topic and who were able to make the expected time investment. In every workgroup, they preferred to have a group leader who coordinated the team, performed role delegation and ensured that deadlines were met. To avoid ambiguities and to facilitate teamwork, they preferred smaller workgroups.

*“Who is included in the working group is somewhat based on interest. So that's just being looked at in the team, who wants to commit to that. We consciously choose to always have a leader that keeps the overview. That ensures that things run smoothly, and that tasks are delegated. We also choose not to make the working groups too large. Because, of course if you are in a working group with 10 people, then no decisions are made.” (GP)*

### Theme 8: How to work patient-centred?

In a patient-centred practice, the patient is invited and empowered to have a steering role in the care process. This means that the patient could, if he wished, be involved in the development of an interprofessional care plan. This enabled the inclusion of the needs and preferences of patients in the care plan. According to care providers, being present at a team meeting could be part of this, but was not necessary. They indicated that care providers were able to represent the patient, though, they did think it was important that information-sharing with the patient was done clearly and strictly.

*“What we regularly do is organize a multidisciplinary meeting. We bring the patient together with all care providers involved in that case. When we feel it is necessary to ensure that everyone is working towards the same goal. […] And the patient is always invited. I think that's an important tool in this story.” (GP)*

Next to medical care, some practices gave attention to providing or if not possible, referring patients to perform (social) activities. More specifically, they designed their practices to be patient-friendly and in some cases, they accepted coaches and specialists from different organisations to support them in person-centred care. However, practices which had no resources or space redirected their patients to external providers.

*“We have something that we call a walk-in cafe, which can be used by several other people who have positive input, such as a coach who provides workshops on creativity, and positivity, … In small, accessible groups to coach persons who are lonely, or from the ‘fourth world’ … making Christmas cards, or smelling or tasting herbs, … This is a sort of a place in which people can participate in a very accessible way to gain information, …” (GP)*

### Theme 9: How to integrate a new team member?

#### Recruiting a new team member

Hiring the right personnel required significant time investment and effort for primary care practices. Overall, care providers were very strict when hiring new team members, and the candidates needed to fit the vision of the practice. They searched for care providers who were able to improve the practice and were ready to invest in caregiving. To do so, questions such as what is good care, how can we improve our practice, and how can you facilitate this improvement, were asked. During the job interview, candidates were informed about the strict requirements, the shared vision and the shared goals of the practice. In some cases, a candidate was not hired if he or she did not fit with the vision of the practice.

*“Anyone who comes to apply for a job with us knows that this is our vision and mission. And we also ask people who apply for a job, 'what do you think we are doing here?' who are the people we see here? How are you going to contribute to make that go better? It's going well, but how is it going to get better? We ask that very consciously. This means that you select people who are willing to invest in it (the practice).” (GP)*

*“We want to offer low-threshold/accessible care. Anyone who comes to apply for a job with us knows that this is our vision. The applicant is asked how he or she will contribute to this. In this way we can select people who want to invest in the high-quality care that we try to offer.”(GP)*

Moreover, in some cases, practices preferred to hire or collaborate with care providers living or who grew up in the neighbourhood. According to care providers, this facilitated the detection of regional/local problems and to work population-centred.

*“Some of them are really local people, from the local community. Our youngest nurse was born and raised in the village. She goes out in the village, knows the people through and through.” (GP)*

Care providers indicated that mastering soft skills is as important as having professional knowledge and skills. When candidates had equal profiles and experiences when hiring new team members, having a flexible and open mindset towards each other was preferred in the practice. Furthermore, they indicated that being open-minded was required to maintain efficient teamwork.

*“But with this employee I helped in the job applications … Actually the three candidates, had the same kind of profile. But where do you start looking for: from whom I think that they can demonstrate a lot of flexibility and an openness towards each other. That is something that is very much needed, a lot of consultation and openness and a lively attitude … So the personality of the people also plays a role in that, in order to be able to build up a collaboration, I think.” (GP)*

Whilst a selection of care providers got involved during the job interviews, the whole team was able to get in touch with the candidate after surpassing the first meeting. In this phase, all team members were involved, asked questions and were included in the final decision.

*“… you don't do an job application procedure with 14 people. There is a fixed structure for it. The vacancy consists of, what profile are you looking for, what are the things that should be seen with it, um. then those application letters are screened by employees of the practice … They are all standard questions. What is your view on healthcare today? And how do you see healthcare in 5 years and in 20 years?… Then the screening was done by everyone (all employees) on Friday.” (GP)*

Besides fitting the vision of the practice, and being open-minded, newly hired caregivers needed to have the capacity and the intention to collaborate. This was required to perform efficient teamwork and to be accepted as “a new colleague.”

*“When selecting […] new employees, we therefore look at the extent to which they agree with our way of working, which is formed by multidisciplinary collaboration. But also whether they have the capacities to work together.” (GP)*

#### Approaching a new team member

According to care providers, just like choosing new team members, integrating new team members was not an easy task. Primary care settings deployed fixed structures (protocols) and strategies to facilitate the integration process of their new colleagues. In some practices, an intake process was organised by one team member (mostly practice assistants or coordinators) in which the new team member learned about the task distribution and the functioning of the practice. Afterwards, (depending on the profile of the new team member) other caregivers joined the in-take process.

*“And then there is actually an intake procedure, in which, when they come, they are explained by the practice assistant about what their part of tasks is and how they should do it. And then (they see) the nurses and then the doctor. he also follows along with everyone, whether you are hired as a physician assistant or as a doctor.” (nurse)*

A caregiver which had recently started within the practice explained how she was integrated in the practice as follows: *“I think as a new employee you are also drawn into this. […] I received those policies before I started working here. Then I was able ask my questions to the coordinator. And then you are directly involved in the story of the things that are now given priority.” (nurse)*

The possibility to follow training based on personal needs and the needs of the primary care settings was experienced as important.

*“But I think that's a very important thing … for further training or at least in certain themes with which I am less confronted with my main job, or how should I say it: where do I still need help, necessary to keep the quality as good as possible within the needs of the practice. I also find it something very important for myself.” (nurse)*

### Theme 10: Getting ready to implement the IPCI toolkit

#### A mix of interventions

According to the participants of the co-design workshops, we need to develop a toolkit with a broad scope, and it should be a mix of interventions. They think that the interventions will not antagonize each other, but it may be beneficial to prioritize some interventions, or to have a chronological order when implementing the interventions. The toolkit should be “dynamic” or adjustable depending on the needs of the practices, care providers and patient. This means that the toolkit is designed in such a way, that it can be strengthened with new tools, without interfering with the existing tool or interventions.

*“Interventions to get to know each other … that seems to me to be the first important building block to start from … Also getting to know each other's expectations, but also being able to properly map out the request for help or the needs from the patient population. Tools/interventions that can emphasize that, are actually very important. And the rest will come naturally. Based on the complexity of the needs (of patients), I think it will always be a mix of interventions.” (WS 7 and 8)*

#### Theory to practice

The co-design workshop participants think that analysing international literature on implementation of interventions is beneficial to provide content for the toolkit. However, they indicated that we should analyse the Flemish context first, to identify the needs, and preferences of practices, care providers, and patients and their families. In addition, these concepts should be adapted in an appropriate way, to the Flemish concept before included in the toolkit. Without this adaption process, they cannot be adopted or implemented successfully.

*“Literature is very important, but when you implement it [the tool], you definitely have to look at the context. First you need to make an analysis of; in which team, in which context do we want an improvement and on what? And then look at how literature can contribute. For me it is important to start from a concrete need (from the local context), and only then look at literature. And not vice versa. …” (WS 3 and 4)*

#### Enabling the implementation of the toolkit

This theme emerged fully from last two sessions of the co-design workshops. Due to an intrinsic motivation, the practices are expected to be more inclined to implement the toolkit properly in their practices. In addition, it was indicated that fewer participants will drop out compared to practices with an extrinsic motivation. This means that practices that benefit from implementing this toolkit will be more likely to continue doing so.

Ownership seems also very important in this context. Practices and caregivers who participate in the implementation of a toolkit should feel that they are part of the project. Their ideas, problems and complaints must be heard. Care providers must be properly guided during the implementation process. Letting them go completely free during a pilot stage was not recommended by the participants. There must be ways (e.g. feedback loops) to maintain contact with the participating practices. Later on, these feedback loops could become part of the toolkit.

The participants of the co-design workshops find coaching in implementing the toolkit very important. Several strategies were mentioned for this. A presentation by the researcher providing more information about the toolkit and interim information moments at the request of the participating teams or individuals could be enlightening and can also facilitate implementation.

## The IPCI toolkit

### Who can use this toolkit?

Based on the study results outlined above, we developed a generic toolkit that can be used by all types of care providers and teams in different primary care settings (41). Both caregivers working under one roof or in close collaboration and caregivers working at different locations can use this toolkit to improve teamwork. The toolkit has eight sections (Table 8), and every module of the toolkit starts with a section in which the concepts and principles used in the toolkit are clarified. All modules are available in a printable PDF format.

**Table 8:** “Building blocks” of the IPCI-toolkit.

Module	Topic	Outcome
1	A self-assessment tool to measure working conditions, psychological safety, interprofessional collaboration, and bio-psychosocial working from the perspective of the care provider.	Measuring: Working conditions, Psychological safety, Interprofessional collaboration, Bio-psychosocial working
2	Preparing care providers to implement the toolkit by teaching them the importance of teamwork and teaching them the basic principles of collaboration	Team readiness and acceptance toward IPCI
3	Teaching care providers the importance and the basic principles of psychological safety.	Changing the attitude of caregivers
4	Consultation techniques: How to prepare for a team meeting? How to organise a team meeting with persons working under one roof? How to develop a network between persons from different settings? How to organise a speed meeting? How to evaluate a team meeting?	Improving different types of team meetings
5	Improving shared decision making: How to deal with concerns/objections from your team members? How to solve the concerns/objections of your team members? You have an agreement, what now? A simple template to document your agreements.	Integrating & implementing shared-decision making in teams
6	Developing workgroups around specific/local problems	Problem-solving. (In setting and regional)
7	Working patient and population centred	Thinking patient-centred.
8	Integrating a new team member	Optimal integration of skills and competences

The modules are introduced with illustrative quotes from patients and caregivers, generated in the development stages. The caregivers can choose which modules they use or not, based on their needs, and preferences. This is necessary since we developed a generic toolkit considering the different contexts of each team as an opportunity to reflect and as a process of identifying problems and solutions. When using the toolkit, the care providers remain in control of the entire care process. The tools we make available serve as a facilitator in collaboration and are designed to guide care providers towards an integrated care. We will now zoom in on each of the sections (Table 8).

### Module 1

A self-assessment tool to measure working conditions, psychological safety, interprofessional collaboration, and bio-psychosocial working from the perspective of the care provider (22, 46–48).

After performing several discussions on how to assess interprofessional collaboration, we concluded that we needed a broad approach. Instead of developing a new measurement tool assessing interprofessional collaboration only, we decided to use a mix of existing, freely available, validated instruments to measure collaboration broadly. First, we will collect sociodemographic data and professional characteristics of the care providers. Afterwards, we measure their health condition, working conditions and job satisfaction (see Table 9).

**Table 9:** Overview of scales to measure sociodemographic characteristics, professional characteristics, health-related questions, working conditions and job satisfaction.

Theme	Source
Sociodemographic characteristics	European Social Survey (49) (adapted to our needs)
Professional characteristics	Primary Care Academy
Health-related questions	European Social Survey (49)
Working conditions and job satisfaction	6^th^ European Working Condition Survey (48)

Secondly, we measure their teamwork through the following three scales: The bio-psychosocial scale (BPSS) (47), the scale for psychological safety (22), assessment of interprofessional team collaboration scale (AITCS) (46), which are listed in Table 10.

**Table 10:** Overview of scales to measure bio-psychosocial working, psychological safety and interprofessional collaboration.

Scales	Source
Bio psycho-social scale (BPSS)	Van de Velde, et al., 2016 (47); De Vriendt et al., 2018 (50)
Scale for psychological safety	Edmondson et al., 1999 (22)
Assessment of interprofessional team collaboration scale (AITCS)	Orchard, et al., 2012 (46)

With the help of these assessment tools, we will map out the situation of care providers and their teams from a broad perspective: to what extent are they and their teams engaged in interprofessional collaboration and integration in primary care.

### Module 2

Preparing care providers to implement the toolkit by teaching them the importance of teamwork and teaching them the basic principles of collaboration.

The toolkit aims to facilitate efficient collaboration between care providers. We learned that to facilitate the implementation of the toolkit, care providers should be prepared by adopting some basic principles of collaboration and teamwork. Based on the interviews and workshops, the following principles of S3 corresponded the most to the caregivers’ needs and preferences. Besides, incorporating the implementation of a toolkit in the goals and vision of settings is an important facilitator to implementing the IPCI-toolkit (see Table 11).

**Table 11:** Basic principles of Sociocracy 3.0, adapted based on data from our research.

Principle	Meaning
Transparency	Make information available for the whole organisation unless it is confidential.
Equality	Involve people when making agreements of evaluations.
Consent	Give, search and integrate objections to decisions and actions.
Accountability	React when it is needed, and take responsibility to keep your organisation on track.
Empiricism	Check all assumptions constantly by experimenting and evaluating your collaboration
Effectivity	Only invest time in those things that bring you closer to achieving your goals.

The following attitude is recommended while performing teamwork of team meetings. Constantly ask yourself the question: Is my behaviour or attitude the most valuable contribution to the effectiveness of this collaboration? This can mean: keeping silent, interrupting, objecting or even breaking agreements.

### Module 3: Enhancing psychological safety

Having a psychologically safe environment seemed to be a precondition to achieving or maintaining efficient teamwork. To improve psychological safety in practices, we introduced a module that includes the following subthemes: (i) be inclusive, (ii) lateral hierarchy, (iii) be open-minded, (iv) have trust, (v) enhance open communication, (vi) be patient, (vii) show respect, (viii) show confidence, and (ix) be comprehensive.

In this module care providers will learn how to enhance an open culture, and they will be able to talk about their problems and mistakes without feeling threatened, which is a precondition for providing “good care.”

### Module 4: Consultation techniques

The care provider longed for structured consultation moments, in which these moments were used optimally in terms of efficiency and effectiveness. Based on the findings of our study, we developed a module incorporating the following subthemes: (i) preparing for a meeting, (ii) performing a basic team meeting with caregivers under one roof, and (iii) building networks between caregivers originating from different settings, (iv) how to organise a speed meeting, (v) Evaluating a team meeting.

In this module, some basic principles are indicated:

- Instead of centralizing all power, it is distributed among the different team members.
- There is a task distribution, which makes it clear who decides what.
- The team members are autonomous, but keep relying on each other.
- The team members’ preferences and their range of tolerance are determined. Between the preferable and the unacceptable lies the tolerance range of humans. By working within this tolerance range, a team can optimize the search for flexibility and perfection.

### Module 5: Shared decision-making and achieving consensus

Healthcare providers who work together must also make decisions together. This can concern decisions about the organization of the practice, patient issues, or other practical matters.

During team meetings, ideas are proposed that may clash with the vision of one or more team members. These disagreements are often resolved quickly, but in some cases can have major consequences for team collaboration. It is important to check whether the objections of the team members are strong enough to count as an objection. In this part, we provide a module to guide caregivers to deal more efficiently with the concerns of team members, make joint decisions and document agreements. A template is provided to document agreements (see attachment). To realize such cooperation, a common language has to be found between the care providers.

In this decision-making process, all parties should be involved, whereby the patient and his/her environment are central.

### Module 6: Developing workgroups to tackle specific (neighbourhood) problems.

Healthcare providers are involved in various processes, inside and outside their setting. Although these processes often go well, problems can occur. Our study shows that many of these problems have already been identified by health care providers, but are not being addressed. These appear to be problems that cannot be solved individually, but which require a team approach. We determined the development of workgroups, which were an effective strategy to tackle specific (local/neighbourhood) problems.

In this module, we provide caregivers with a five-step approach to tackle these care or neighbourhood problems, starting from: you have identified a problem and you understand that you cannot solve this problem on your own. How are you going to handle this?

The five steps:

- First, check if a team member is already working on solving that problem.
- Find out who is involved in, and/or affected by this issue.
- Make yourself a shortlist of colleagues who may be able to participate.
- Motivate your colleagues to participate by explaining what’s in it for them.
- What do your colleagues expect from you and what contribution can you expect from them?

Each of these steps is further explained in the IPCI-toolkit.

### Module 7: How to work person-centred and population-centred?

Person-centred care is treating a person/patient in an honourable and respectful manner, and involving hem in all decisions made in the care he/she receives.

By working in a more person-oriented way, the caregiver can provide better care to the patient and his/her environment. According to caregivers, patients expect the caregiver to see them as a partner in care and hope that their needs, preferences and experiences will be taken into account. Giving patients a say in the care and treatment they receive, can be beneficial for their care process. As a result, patients will be better informed and have an improved adherence to the therapy. In addition, a better relationship between the healthcare provider and patient will be achieved.

We provide a module for caregivers to collaborate with the patient and his/her family and environment.

### Module 8: How to integrate a new team member?

In this module, we developed seven steps to guide practices in the integration of a new team member: (i) preparation for the arrival of a new colleague, (ii) welcoming a new colleague, (iii) taking initiative and introducing the new team member, (iv) clarifying your team’s vision, values, goals and priorities, (v) explain how the roles and associated responsibilities are distributed, (vi) take advantage of the lunch breaks, (vii) make it clear that the new team member can contact any caregiver with all his/her questions.

## Discussion

The two-year development process resulted in a 38 paged, generic, Dutch toolkit. It is a manual adapted to the concepts and framework of Sociocracy 3.0 and psychological safety. It consists of eight modules: (i) self-assessment tool, (ii) improving team readiness and acceptance towards the use of a toolkit, (iii) improving psychological safety, (iv) consulting techniques, (v) shared decision making, (vi) developing working groups around specific problems, (vii) how to work person and population centred, and (viii) how to integrate a new team member. The toolkit intents to help caregivers coordinate their care and improve the communication between various health actors, patients, (in) formal caregivers, and families.

Self-evaluation was considered a way to assess team performance and to identify specific issues on collaboration and team integration. By providing them the right assessment tools, care providers can identify their shortcomings, detect areas for improvement and start looking for solutions. Moreover, identifying the issues regarding collaboration were preconditions for better teamwork. We found that the main influential factors were (i) the understanding of the necessity of interprofessional collaboration, in agreement with Reeves et al. (37), and (ii) the explicit presence of shared vision and goals, in agreement with Johnson et al. (51). Our research also revealed this shared vision should be revised periodically. These updates should reflect the evolving needs and preferences of the practice and its care providers incorporating the views of patients and their families. However, this is not included in the tool?

In our study, psychological safety, having a safe team climate, helped care providers to achieve a lateral hierarchy, to have trust in each others’ competencies, and to have an open culture. According to Edmondson et al. (23), this psychologically safe environment is a prerequisite for teamwork and Dieckmann et al. (52) add that it facilitates practice innovation. In our study, the psychological safety of patients and/or their families during consultations or treatments was not explicitly mentioned in contrast to the findings of Hunt et al. (53) in mental health services.

Inspired by the Sociocracy framework, a variety of consultation, and decision-making techniques have incorporated such as formal and informal meetings, speed meetings, meetings with various organizations/practices, and conflict resolution techniques. During these meetings, the organisation of the practice, patient issues, or other practical matters were discussed. Although care providers agreed that the patient should be considered as a full partner, they were not present at these meetings. Van Dongen et al. (54) indicated that patient participation in interprofessional team meetings was appreciated by professionals and patients, however, support and readiness for the meetings was needed. According to Rollet et al. (55), patient participation was associated with better treatment, longer survival, improved trust and compliance with the treatment (56). Our research presented that the patient participation could be strengthened by having a patient-centred care approach, where the patient is treated in a sincere and respectful manner, and in which the patient is involved in all decisions made in the care he/she receives.

To tackle specific or local problems, care providers in our study indicated they sometimes use workgroups. This development process started with first identifying the problems occurring in and around their practice. Secondly, they searched for suitable team members who were capable of solving these problems. They also facilitate population-based working, which is, according to Kringos et al. (5) relevant for prevention and a more integrated collaboration within the public health sector.

Currently, practices are facing a shortage of healthcare workers and fragmentation. Moreover, new team members experience various issues and inefficiencies while integrating in the team. To avoid these issues, and to optimize the use of resources, we introduced, inspired by Ellis et al. (57), seven steps to guide and support practices in the integration of a new team member.

This study has several strengths which will be explained in this paragraph. We ran a bottom-up multi-staged trajectory, including the views and opinions of more than 120 practitioners, professionals, academics, and patient representatives. Performing a combination of in-depth interviews and focus groups in general practices, and mental health organisations, allowed us to collect data from different types of professionals, working in different types of primary settings. Subsequently, the co-design workshops gave us the opportunity to analyse and evaluate our findings with a larger group, and it allowed us to maintain an interprofessional approach while analysing and evaluating our findings. We chose to use triangulation, as many researchers and practitioners of different backgrounds and primary care settings analysed and evaluated our findings along the way. This reduced the risk of bias and added to a broader applicability of the toolkit.

There are several potential limitations to this study. Given the complexity of interprofessional collaboration and the changing environment in primary care, this toolkit may not have covered all issues in the broad context of Flemish primary healthcare. In addition, since all data is collected in primary care settings in Flanders and mostly with care providers working in a mono or multidisciplinary group practices. Therefore, the findings may not be generalizable to other regions, other levels of healthcare and solo practices. The literature has established that researchers can influence the interpretation of data, and despite our methodology in which we made efforts to reduce bias, it is common in qualitative research that the presence of a researcher influences the interpretation of the data (6). To address this problem, Lincoln and Guba (1985) indicated the following four general criteria in their approach to trustworthiness: credibility, transferability, dependability, and confirmability (58, 59). Since our self-evaluation tool is directed to care providers, this might not enable the identification of problems experienced by patients or clients. Validated instruments such as Patient Reported Experience Measures (PREMS) (60, 61) and Patient-Reported outcome Measures (PROMS) (62, 63), are available but these were never used by the care providers who participated in our study. However, Black et al. (64) indicated that the use of these measures could help with transforming practices, and Wolff et al. (65) mentioned that it facilitated patient-centred care. Next to limitations, this study has also several strengths. This risk of bias was minimised by triangulating researchers from different backgrounds (e.g. nurses, pharmacists and a psychologist) trough the whole process. This triangulation, intensive cooperation and inductive process increased the credibility and reduced the risk of bias to the interpretation of the data based on preconceived understanding and personal opinions. Previous to this research, a literature review of existing strategies and interventions was performed by the same researchers. This ensured that the researchers were aware of existing strategies, toolkits and interventions so they made use of them. Unlike the interventions identified in this literature review, the development process, research data is provided in this paper, and the full toolkit is attached as an appendix. In addition, by organising co-design workshops with a very broad group, the researchers were able to develop a toolkit that takes into account multiple perspectives.

Though very complex in nature and sometimes difficult in practice, interprofessional collaboration seems to be a prerequisite for integrated care. It benefits quality of care when it is based on the needs and preferences of practices, care providers, and patients and their families. We expect that this toolkit will need to be adapted, improved, as well as extended in the coming years, based on the changing landscape of primary care or new insights gained form more research. Hereby, a new study is set up by the same research team to evaluate the usability and efficacy of the toolkit, and subsequently modify the toolkit based on the research findings.

## Conclusion

In this paper, we describe the multiyear co-development process of a generic toolkit for the improvement of interprofessional collaboration. Inspired by a mix of interventions from in and outside healthcare, a modular open toolkit was produced that includes aspects of Sociocracy, concepts as psychological safety, a self-assessment tool and other modules concerned with meetings, decision making, integrating new team members and population health. Upon implementation, evaluation and further development and improvement, this compounded intervention should have a beneficial effect on the complex problem of interprofessional collaboration in primary care.

## Data availability statement

The raw data supporting the conclusions of this article will be made available by the authors, without undue reservation.

## Ethics statement

Approval for this study was obtained from the Ethical Committee of Antwerp University Hospital with the file number 20/38/477. The study was in accordance with the principles outlined in the Declaration of Helsinki. The participants received verbal and written information about the purpose and methods of the study and written informed consent was approved by the above-mentioned Ethical Committee. Participants were informed that participation was voluntary, and that confidentiality would be ensured.

## Author contributions

MMS, HDL, KdV, KVdB, and PVB: development of the research question, establishment of the research strategy, data collection, and data analysis. MMS, HDL, KdV, KVdB, PVR, PP, KD, EV, RR, and PVB: discussion construction and writing-review and editing. MMS and PVB are the guarantors of this research. All authors contributed to the article and approved the submitted version.

## Funding

This work was funded by the King Baudouin Foundation and Fund Daniel De Coninck (Grant number: 2019-J5170820-211588).

## Conflict of interest

The authors declare that the research was conducted in the absence of any commercial or financial relationships that could be construed as a potential conflict of interest.

## Publisher’s note

All claims expressed in this article are solely those of the authors and do not necessarily represent those of their affiliated organizations, or those of the publisher, the editors and the reviewers. Any product that may be evaluated in this article, or claim that may be made by its manufacturer, is not guaranteed or endorsed by the publisher.
