# Supplementary material for: Development of a toolkit to improve interprofessional collaboration and integration in primary care using qualitative interviews and co-design workshops
Source: Front Public Health. 2023 Apr 17;11:1140987. doi: 10.3389/fpubh.2023.1140987 (PMC10149845; doi:10.3389/fpubh.2023.1140987)
Supplement: Supplementary file 1 [file Data_Sheet_1.PDF]

Een toolkit voor een betere samenwerking en integratie in de eerste lijn

Auteurs: Muhammed Mustafa Sirimsi (PhD student), Prof. Dr. Hans De Loof, Prof. Dr. Kris Van Den Broeck, Dr. Kristel De Vliegher, Prof. Dr. Peter Van Bogaert

Academie Voor De Eerste Lijn

Thuis in de eerste lijn
FONDS DR. DANIËL DE CONINCK

Koning Boudewijnstichting
Samen werken aan een betere samenleving

Gelieve dit document niet te delen zonder toestemming van de auteurs.

Inhoud

1. Zelfevaluatie......6
2. Hoe bereid je een team voor op het gebruik van deze toolkit? 7
3. Psychologische veiligheid bevorderen......11
4. Overlegtechnieken......15
4.1. Hoe bereid je een teamoverleg voor?......16
4.2. Overlegmoment met zorgverleners onder één dak......18
4.3. Hoe bouw je een netwerk tussen zorgverleners uit verschillende organisaties? 20
4.4. Organiseren van speed meetings......22
4.5. Hoe evalueer je een team-meeting?......25
5. Gezamenlijke besluitvorming......26
5.1. Hoe ga je om met bedenkingen van je teamleden?......27
5.2. Hoe los je conflicten of bezwaren van je collega's op?......28
5.3. Je hebt een akkoord bereikt met je collega's, wat nu? 29
6. Hoe stel je een team (werkgroep) samen rond een specifieke problematiek......31
7. Hoe werk je persoonsgericht? 33
8. Hoe integreer je een nieuwe teamlid? 34
Bijlagen......36

Inleiding

Doel

Binnen de eerstelijnszorg is er een toenemende behoefte aan methodieken, technieken en structuren die effectieve samenwerking ondersteunen.

In deze toolkit krijgen zorgverleners hulpmiddelen (tools) aangereikt met het oog op het verhogen van de productiviteit van het teamwerk, alsook om de interprofessionele samenwerking en integratie in de eerste lijn te faciliteren. Dit houdt in dat we zorgverleners helpen bij het communiceren en coördineren van zorg met en tussen verschillende actoren (o.a. patiënten, (in)formele zorgverleners, mantelzorgers, families,…)

De toolkit bestaat uit acht onderdelen en is een synthese van uit wetenschappelijke literatuur gefundeerde concepten, die op basis van ervaringen van patiënten en zorgverleners zijn gefinetuned. Concreet bieden we zorgverleners een handleiding aan waaruit men, op basis van de eigen noden en behoeften, diverse strategieën kan kiezen ter ondersteuning en ter versterking van de samenwerking.

De acht onderdelen zijn:

1. Zelfevaluatietool
2. Hoe bereid je een team voor op het gebruik van een toolkit?
3. Psychologische veiligheid bevorderen
4. Overlegtechnieken
5. Gezamenlijke besluitvorming
6. Hoe stel je een team (werkgroep) samen rond een specifieke problematiek?
7. Hoe werk je persoonsgericht?
8. Hoe integreer je een nieuw teamlid?

Voor wie?

Deze algemene of generieke toolkit is bruikbaar voor alle type zorgverleners, teams en zorginstellingen in de eerstelijnszorg. Zowel zorgverleners die onder één dak werken, als zorgverleners op verschillende locaties en niveaus kunnen deze toolkit gebruiken om hun samenwerking te versterken.

Hoe kwam de toolkit tot stand?

Deze toolkit is het resultaat van wetenschappelijk onderzoek uitgevoerd in een doctoraatsproject. We doken enerzijds in de literatuur. Daaruit weerhielden we waardevolle concepten, werkwijzen en strategieën die we in deze toolkit als bouwstenen inbrachten. Anderzijds bevroegen we de ervaringen, noden en voorkeuren van zorgverleners, patiënten en mantelzorgers, specifiek over het werk in de Vlaamse eerstelijnszorg, met (groeps)interviews en enquêtes. Het resultaat is een combinatie van internationale wetenschappelijke evidentie met pragmatische ervaringskennis, -wensen en –behoeften uit de dagdagelijkse Vlaamse praktijk.

Over het gehele ontwikkelingsproces werd er bijkomend gebruik gemaakt van de expertise van een academisch team dat zich heeft ingezet om deze toolkit operationeel te maken.

Hoe werkt de toolkit?

Elk onderdeel van de toolkit start met een info-sessie waarin de gebruikte principes en concepten van de toolkit kort worden toegelicht. Deze toelichting is ook beschikbaar op de website https://academievoordeeerstelijn.be/interprofessionele-samenwerking/ in de vorm van een filmpje of handleidingen. Alle tools zijn beschikbaar in Pdf-formaat en kunnen afgedrukt worden. (Dit is tijdens de piloot-fase niet van toepassing)

De tools worden ingeleid met getuigenissen van patiënten en zorgverleners, gebaseerd op de interviews en bevragingen tijdens het onderzoeksproces.

Om gericht rond bepaalde problematieken te werken, worden er eerst verbeterpunten geïdentificeerd via de zelfevaluatietool. Op basis van deze resultaten kunnen zorgverleners hun sterktes en zwaktes te weten komen en kunnen verschillende gestructureerde samenwerkingsstrategieën en overlegvormen uitvoeren die beschikbaar zijn in de toolkit.

Bij het gebruik van de toolkit blijven de zorgverleners in regie over het gehele zorgproces. De tools die we beschikbaar stellen, dienen als facilitator/ hulpmiddel in de samenwerking en integratie in de eerstelijnszorg.

1. ZELFEVALUATIE

Om de eerstelijnszorg de plaats te kunnen geven die ze verdient – centraal in ons zorgsysteem – is het belangrijk om zicht te krijgen op uw werkomstandigheden: bv. Jobtevredenheid en gezondsgerelateerde problemen.

In dit onderdeel willen we met behulp van een meetinstrument de situatie van u en uw team in kaart brengen: in welke mate wordt er door u en uw team aan interprofessionele samenwerking en integratie gedaan. Dit houdt in dat we bijvoorbeeld vragen naar: hoe werkt u samen met andere zorgverleners en hoe ondersteunt u patiënten/cliënten. Daarnaast wordt er rekening gehouden met de noden van zorgverleners, die we - samen met jullie – in kaart kunnen brengen en aanpakken.

Inhoud vragenlijst:

1. *BLOK 1: Demografische gegevens eerstelijnsprofessional*
2. *BLOK 2: Professionele gegevens eerstelijnszorgverstrekkers*
3. *BLOK 3: Gezondheidsgerelateerde vragen*
4. *BLOK 4: Werkomstandigheden en jobtevredenheid*
5. *BLOK 5: Schaal voor psychologische veiligheid*
6. *BLOK 6: AITCS*
7. *BLOK 7: BPS*

ALGEMEEN

- *Elke vraag moet ingevuld worden, anders kan men niet door naar de volgende.*
- *Telkens mag maar één antwoord aangeduid worden, tenzij anders vermeld.*

**Zie bijlage voor de zelfevaluatietool.*

2. Hoe bereid je een team voor op het gebruik van een toolkit?

Het doel van de toolkit is de samenwerking tussen zorgverleners op een efficiënte wijze te faciliteren. Om de implementatie van de toolkit te vergemakkelijken, is het aangeraden de gebruikers voor te bereiden door hen een aantal competenties aan te leren.

De 7 basisprincipes en vier competenties die de zorgverleners dienen te beheersen voor een goede samenwerking, worden hieronder aangeven.

De zeven basisprincipes:

1. Transparantie: Maak alle informatie beschikbaar voor iedereen in de organisatie, tenzij er een reden is voor vertrouwelijkheid
2. Gelijkwaardigheid: Betrek mensen bij het maken en evalueren van afspraken die hen raken
3. Consent: Geef, zoek en integreer bezwaren tegen beslissingen en acties
4. Continu verbeteren: Breng continu kleine veranderingen aan om empirisch leren mogelijk te maken
5. Accountability: Reageer wanneer iets nodig is, doe waar je mee hebt ingestemd en neem verantwoordelijkheid voor de koers van de organisatie
6. Empirisme: Test alle veronderstellingen door voortdurend te experimenteren en te evalueren
7. Effectiviteit: Investeer alleen tijd in die dingen die je dichter brengen bij het bereiken van je doelen

Stel jezelf geregeld de vraag: “Is mijn gedrag* op dit moment de meest waardevolle bijdrage aan de effectiviteit van deze samenwerking?”

**betekent mogelijk ook zwijgen, onderbreken, bezwaar uiten of zelfs afspraken verbreken*

2.1 Basisprincipes en competenties voor een goede samenwerking

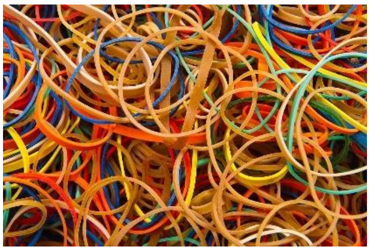

Wendbaarheid: Als zorgverlener is het van belang snel en effectief te reageren op veranderingen in je omgeving. Hiervoor moet je eerst te weten komen wat deze veranderingen zijn en is het belangrijk om de juiste mensen te vinden voor de nodige veranderingen. Op deze manier is je organisaties waterdicht en voorbereid op crisissituaties.

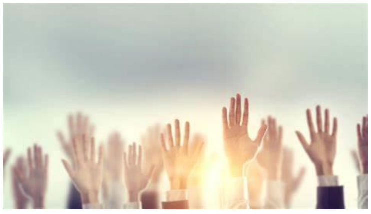

Participatie: Als zorgverlener en gezondheidsprofessional ben je verantwoordelijk voor je eigen ontwikkeling en wordt er van je verwacht dat je participeert in teamwerk. Hierbij wordt er van je verwacht dat je je competenties maximaal benut. Zorg ervoor dat je collega's op je kunnen rekenen als ze je nodig hebben.

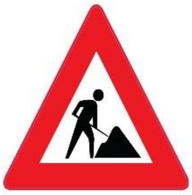

Uitvoering: Als zorgverlener heb je je handen vol aan dagdagelijkse taken. Het kan soms moeilijk zijn om hier bovenop overlegmomenten in te plannen, je collega's bij te staan … Daardoor is het belangrijk om keuzes te maken en prioriteiten te stellen op basis van je ervaringen.

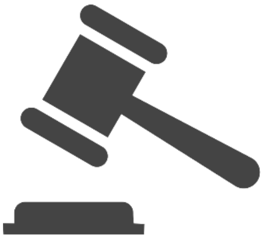

Besluitvorming: Beargumenteer je redenering als je iets voorstelt. Betrek hierbij je collega's en wees niet bang voor kritiek. Door elkaar bij te staan en te verbeteren worden jij en je team alleen maar sterker.

Werkwijze:

1. Zorg ervoor dat ieder teamlid wordt betrokken.

Elk teamlid:

- begrijpt waarom verandering nodig is.
- is aanwezig wanneer er beslissingen worden genomen.
- kent het opzet van de tools.
- gebruikt de tools op een juiste manier.

Experimenteer voldoende met de toolkit, zodat de teamleden de voordelen en mogelijke valkuilen van de toolkit begrijpen.

2. Creëer een omgeving die veranderingen en invoering van deze toolkit mogelijk maakt.

- Gebruik tools die oplossingen bieden voor de problemen die je hebt geïdentificeerd.
- Stop niet met hetgeen dat werkt.
- Blijf niet proberen als je beseft dat een invoering niet werkt in je praktijk.
- Laat de teamleden op eigen tempo werken.

3. Be the change

Wees een voorbeeld voor je team tijdens dit veranderingsproces. Handel en gedraag je op een manier, zoals je wilt dat de anderen zich gedragen.

4. Nodig je collega's uit voor verandering in plaats van ze te verplichten

Maak duidelijk waarom je iets wilt veranderen aan de huidige gang van zaken en nodig je collega's uit om te participeren aan deze verandering.

Door je collega's uit te nodigen in plaats van ze te verplichten, verminder je de kans op tegenwerking en geef je de teamleden het – terechte – gevoel dat ze een deel zijn van de beslissing.

Enkele tips:

- Wees transparant over de reden voor verandering
- Maak duidelijk wat je verwacht van deze verandering
- Geef aan welke beperkingen en valkuilen je voorspelt.
- Voorkom dwang of manipulatie.
- Houd rekening met mogelijke twijfels en vragen.

6. Includeer collega's die een voordeel halen uit het gebruik van deze toolkit.

Hierdoor verhoog je de betrokkenheid en interesse van de deelnemende collega's en worden de tools beter benut.

Selecteer collega's die:

- zoeken naar een hulpmiddel voor een betere samenwerking.
- regelmatig aangeven moeite te hebben met coördinatie van zorg.
- regelmatig overleggen met mensen binnen- en buiten de praktijk.

7. Creëer ruimte voor verandering

Nodig uit om mee te werken en te experimenteren met de toolkit. Geef hun een plek in de evolutie van de praktijk.

- Maak duidelijk wat je driver is voor deze verandering
- Plan enkele momenten in om de toolkit gezamenlijk uit te testen. Beschouw de tijd die je hierin steekt als een investering en niet als een last.
- Reflecteer op deze testmomenten en leer ervan.

3. Psychologische veiligheid bevorderen

Of je nu werkt in een internationaal bedrijf, een KMO of een praktijk in de gezondheidszorg, als kenniswerker kom je ongetwijfeld in contact met verschillende types organisaties of mensen die deel uitmaken van een organisatie. Deze interactie gaat vaak gepaard met het gebruik van verschillende samenwerkingsvormen, waarin psychologische veiligheid van cruciaal belang is.

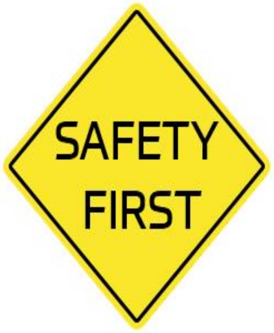

Een psychologisch veilige omgeving bekomen, is een voorwaarde voor een efficiënte communicatie en samenwerking in de eerstelijnszorg. Om dit te verwezenlijken worden tools aangeboden die de zorgverleners kunnen gebruiken om hun samenwerking te versterken.

Getuigenis zorgverlener:

ZV: "Om een open cultuur te hebben moet je vooral een veilig gevoel hebben."

ZV: "bijvoorbeeld als ik een fout maak, dan moet ik dat altijd kunnen zeggen, gelijk tegen wie, gelijk wat ik heb gedaan, dat is een basisvoorwaarde van goede zorg"

Wat bieden we aan?

In dit onderdeel bieden we enkele handvaten aan die teams kunnen gebruiken voor een attitude die een psychologisch veilig omgeving faciliteert.

Mogelijke valkuilen:

- Het is belangrijk dat ieder teamlid begrijpt wat psychologische veiligheid inhoudt en welke voordelen dit met zich meebrengt.
- De toolkit is een hulpmiddel om gestructureerd te communiceren, maar dit mag geen barrière vormen voor het voeren van informele gesprekken.
- Indien er een sterk hiërarchische structuur aanwezig is, kan het uitvoeren van interventies rond psychologische veiligheid grote problemen veroorzaken binnen de teams
- Het is belangrijk dat ieder teamlid wordt betrokken in het verhaal.
- Psychologische veiligheid mag geen middel zijn om de eigen agenda door te drukken in de praktijk.

Handvaten om psychologische veiligheid te bevorderen:

**Toon interesse**
Wees alert en focus op de conversatie. (vb. Kijk naar je collega, leg je smartphone of laptop opzij, …)
Stel vragen met de intentie om bij te leren van je collega's.
Geef input, wees interactief en maak duidelijk dat je aan het luisteren bent.
Antwoord verbaal en toon interesse. (vb. Dat klinkt goed, Vertel daar eens meer over, …)
Houd rekening met je lichaamstaal; begeef of draai je met je gezicht naar je collega die spreekt
Maak oogcontact om te duidelijk te maken dat je luistert en belang hecht aan wat je collega te vertellen heeft.

**Toon begrip**
Vat geregeld samen wat je collega's hebben verteld om duidelijk te maken dat je hebt geluisterd en begrijpt wat ze hebben verteld. (vb.) Maak ook duidelijk waarmee je het eens of oneens bent en sta open voor mogelijke vragen die ze hieromtrent kunnen stellen. (vb. Als ik het goed begrijp, bedoel je …)
Bevestig verbaal dat je akkoord gaat met en/of begrijpt wat er wordt gezegd. (Ik begrijp wat je bedoelt, knikken terwijl je collega spreekt, …)
Vermijd beschuldigingen vb. waarom heb je dit gedaan, …) en focus op het zoeken van oplossingen. (vb. wat kunnen we doen, om deze problemen te voorkomen, hoe kunnen we dit nu oplossen,…)
Controleer je (on)bedoelde gezichtsuitdrukkingen. (Probeer fronsen, oogrollen, … te vermijden)

Probeer geregeld te knikken tijdens een conversatie op momenten dat je akkoord gaat met wat je collega's vertellen.

**Wees inclusief tijdens interpersoonlijke relaties**
Deel informatie over je persoonlijke werkstijl, noden en voorkeuren. Motiveer je collega's om dit ook te doen.
Wees bereikbaar en laagdrempelig voor je collega's. (vb. maak tijd voor adhoc één op één conversaties (ook informeel), coaching en feedback sessies)
Communiceer op een heldere manier de reden van de meetings die je wilt organiseren.
Toon dankbaarheid voor de inspanningen en bijdrage van je teamleden.
Grijp in wanneer teamleden negatief praten over een ander teamlid. (vb. roddelen, lobbyen, …)
Vertoon een open lichaamshouding tegenover je collega's. (vb. kijk naar al je collega's tijdens een gesprek, niet met je rug keren naar een deel van je team, …)
Bouw aan een vertrouwensrelatie. (vb. spreek met je teamleden over hun leven buiten het werk, …)

**Wees inclusief bij het nemen van beslissingen**
Vraag actief naar input, meningen en feedback van je collega's.
Onderbreek je collega's niet en sta dit ook niet toe tijdens de overlegmomenten. (Zorg ervoor dat alle teamleden hun mening kunnen uiten zonder onderbroken te worden)
Verklaar je beslissing en geef achtergrondinformatie hoe je komt tot deze beslissing. (Dit kan mondeling, via e-mail, …)
Accepteer en erken de input van anderen. (vb. geef erkenning aan de betrokken teamleden bij een beslissing of succes).

Toon zelfvertrouwen en overtuiging zonder eigenwijs over te komen
Spreek klaar en duidelijk in een teamgesprek. (voldoende luid, goede articulatie, …)
Steun en representeer je team.
Nodig je team uit om feedback te geven op je beslissingen of suggesties en ga hierop in. In discussie gaan kan leiden tot innovatie en verbetering.
Toon je kwetsbare en mindere kanten. Deel je persoonlijke (goede en slechte) ervaringen, geef je persoonlijke mening, …
Motiveer je teamleden om risico’s te nemen en doe dit ook zelf.
Probeer discussies tussen je teamleden te managen. (vb. sta geen meerdere onderlinge conversaties toe, maak duidelijk dat onenigheden niet persoonlijk maar rond een bepaalde topic zijn,…)

4. Overlegtechnieken

Uit onderzoek blijkt dat een psychologisch veilige werkomgeving de organisatie van team-meetings faciliteert. De zorgverlener verlangt naar gestructureerde overlegmomenten, waarbij deze momenten op vlak van efficiëntie en effectiviteit optimaal worden benut.

Getuigenis zorgverlener?

Vraag: *"Wat als de inhoud van die overlegmomenten iets structureler zou zijn?"*

Antwoord: *"… het zou de efficiëntie ontzettend veel kunnen verhogen en ja, het geklungel van wat wij nu bij elkaar hebben proberen te mixen … dat wij dat niet meer moeten doen en dat wij gewoon inhoudelijk ons expertise daarin kunnen brengen. Dus ik denk dat het heel handig is als ge daar ook extern iemand voor hebt."*

Enkele basiselementen:

- In plaats van alle macht te centraliseren, wordt deze verspreid onder de verschillende teamleden.
- Er vindt een verdeling plaats, waardoor het duidelijk wordt wie wat beslist.
- De teamleden zijn autonoom, maar blijven wel afhankelijk van elkaar.
- De voorkeuren van de teamleden en hun *tolerantiebereik** wordt bepaald.

In dit onderdeel stellen we enkele handvaten ter beschikking die teams kunnen gebruiken om hun overlegmomenten gestructureerd uit te voeren. Echter, inhoudelijk worden deze overlegmomenten vrij ingevuld door de zorgverleners zelf.

Inhoud:

- Hoe bereid je een overlegmoment voor?
- Overlegmoment met zorgverleners onder één dak
- Hoe bouw je een netwerk tussen zorgverleners uit verschillende organisaties?
- Organiseren van speed-meetings
- Hoe evalueer je een team-meeting?

*Tolerantiebereik: Tussen de voorkeur en het onaanvaardbare ligt het tolerantiebereik van mensen. Door te werken binnen dit tolerantiebereik, kan een team de zoektocht naar flexibiliteit en perfectie optimaliseren. (ik zou deze definitie naar onder brengen, dan staat het vlak naast het eerste gebruik van de term)

4.1. Hoe bereid je een teamoverleg voor?

Een goede voorbereiding is cruciaal om een effectief en efficiënt teamoverleg te ervaren. Om deze voorbereiding te faciliteren worden agenda's opgesteld die de zorgverleners voor de aanvang van de meeting kunnen inkijken.

Daarnaast kunnen voor de start van de meeting bepaalde rollen verdeeld worden onder de teamleden.

Getuigenis zorgverlener:

"…dat werd dan ook voorbereid door de verpleegkundigen. Dus als verantwoordelijke voor de diabetespatiënten ging ik dan alles oplijsten van wat er moest gebeuren…"

Elk teamlid handelt semi-autonoom. Dit betekent dat:

- Een teamlid handelt binnen de beperking van zijn/haar eigen domein
- Deze teamleden zullen voortdurend samen beslissingen nemen om aan hun eigen noden en voorkeuren te voldoen en te bespreken welk stappenplan ze hiervoor nodig hebben.

Daarnaast is elk teamlid evenveel verantwoordelijk voor het bestuur en de beslissingen die worden genomen binnen het team.

Rolverdeling tijdens overleg:

1. Secretaris: Onderhoudt alle documenten die te maken hebben met overlegmomenten.
2. Facilitator: Zorgt ervoor dat meetings vlot verlopen en dat iedereen in de meeting zeggenschap heeft.
3. Afgevaardigde: Zorgt ervoor dat info wordt gedeeld met zorgverleners buiten de cirkel.
4. Leider/coördinator: Zorgt ervoor dat alles volgens de visie van de organisatie verloopt en dat de einddoelen worden behaald.

In een team met meer dan vier leden krijgt niet iedereen een rol toebedeeld. Deze teamleden krijgen operationele rollen die ad hoc worden bepaald, volgens de noden van de opdrachten. Een teamlid kan meer dan één rol op zich nemen.

Hoe stel je een agenda op?

Hoe bereid je je voor als participant?

Hoe duid je een facilitator aan?

Werkwijze:

Hoe stel je een agenda op?

Maak een agenda klaar voor de meeting. Doe dit niet alleen, maar in team. Help je collega's eventueel bij hun voorbereiding. Gebruik hiervoor de template in Bijlage X.

Beslis samen:

- Wat de driver van de meeting is.
- Wat je verwacht van de meeting.
- Hoe deze meeting zal verlopen.
- Hoe lang de meeting zal duren en hoeveel tijd het inneemt om dit voor te bereiden.
- Wat de participanten moeten doen om zich voor te bereiden.

Hoe bereid je je voor als participant?

Als participant moet je:

- De agenda op voorhand bekijken en jezelf voorbereiden op de topics.
- Een lijst maken met zaken waarmee je niet akkoord bent.
- Verbeteringen suggereren op basis van de vorige vergadering(en).

Hoe duid je een facilitator aan?

Duid iemand aan die:

- Overleg vlot laat verlopen.
- Zorgt dat iedereen aan bod komt.
- Zorgt dat teamleden zich focussen op het agendapunt dat aan de orde is.
- De tijd in het oog houdt.
- Op het einde van de meeting een korte samenvatting geeft.

4.2. Overlegmoment met zorgverleners onder één dak

Zorgverleners die onder één dak werken, organiseren geregeld overlegmomenten. Dit gebeurt in verschillende formats en met een variërend aantal en type zorgverleners.

Hoewel de patiënt niet is betrokken in deze overlegmomenten, zien we dat inefficiënte communicatie en zorgcoördinatie onder zorgverleners resulteert tot ongenoegen bij de patiënten.

Getuigenis van de patiënt:

De patiënt wordt door verschillende hulpverleners behandeld en vindt het belangrijk dat de zorg op elkaar is afgestemd. Het maakt voor de patiënt echter niet uit hoe de zorg gecoördineerd wordt, zolang er maar coördinatie is.

Getuigenis van de zorgverlener:

Zorgverleners erkennen het belang van communicatie en zorgcoördinatie. Alles begint voor hen met een duidelijk overzicht van wie welke functie binnen het netwerk uitoefent. Het samenwerken is geen gemakkelijke opdracht, maar wel een noodzakelijke opdracht. Als team sta je sterker dan alleen.

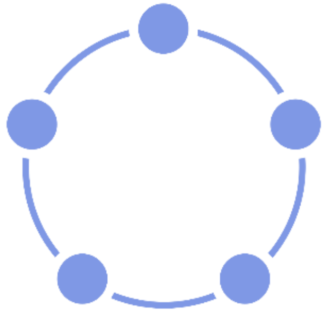

Een enkele cirkel

Wat bieden we aan?

In dit onderdeel worden handvaten aangeboden om met behulp van een unieke en gestructureerde tool een overleg te houden met je vaste teamleden.

Enkele begrippen:

- <u>Cirkel:</u> Elk team wordt voorgesteld als een cirkel, die semi-autonoom is. In deze cirkel wordt alle macht verspreid onder de verschillende teamleden, i.p.v. ze te centraliseren bij één iemand.
- <u>Rondes:</u> De teamleden communiceren in rondes. Dit betekent dat iedereen aan bod komt en ongeveer evenveel tijd krijgt om te spreken over een topic. Laat de rondes telkens met een ander teamlid beginnen.

Door rondes in te voeren, geef je de teamleden het gevoel dat alle ideeën even belangrijk zijn en zorg je ervoor dat ieder teamlid aandachtig luistert of voorbereid in de vergadering komt.

Werkwijze:

Welke stappen moet je ondernemen?

1. Zit in de vorm van een cirkel rond de tafel.
2. Werk in rondes en geef in de meeting ieder teamlid de kans om te spreken.
3. Begin en eindig elke ronde met een ander teamlid.
4. Bespreek één topic per ronde.
5. Las indien nodig meerdere rondes in voor één topic.

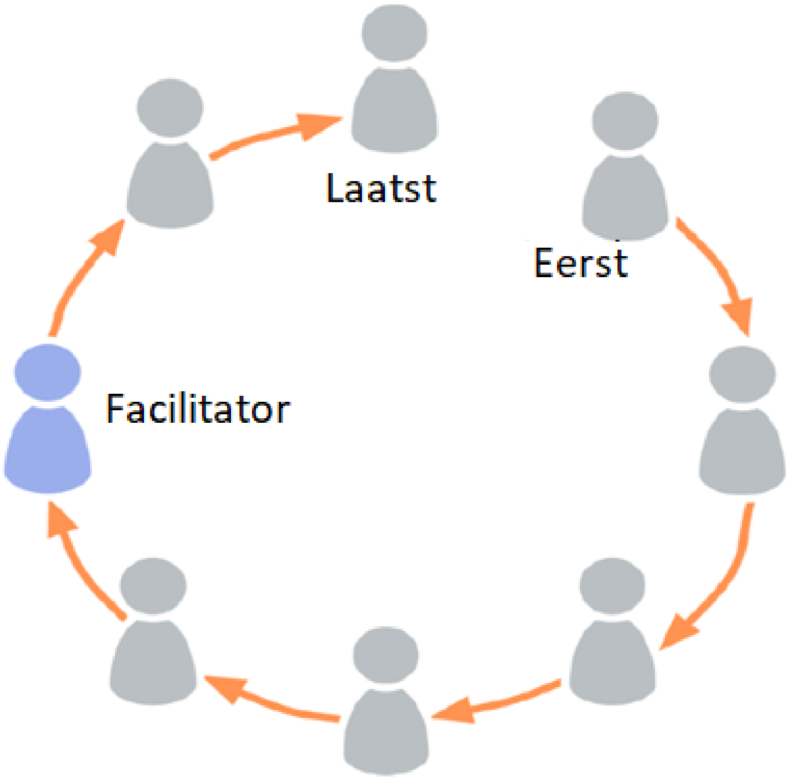

Structuur voor overlegmoment tussen zorgverleners

4.3. Hoe bouw je een netwerk tussen zorgverleners uit verschillende organisaties?

Met behulp van deze tool kunnen praktijken/teams efficiënter met zorgverleners uit verschillende organisaties samenwerken. Dit houdt in dat verschillende teams gemakkelijker informatie uitwisselen, meetings organiseren en samen beslissingen kunnen nemen.

Getuigenis van de patiënt:

"Zorgverleners moeten durven om hun kijk te verbreden en verder te kijken dan hun eigen discipline. Ze moeten openstaan om contact te hebben met andere zorgverleners zodat ze 'één' worden. Zo zou de patiënt ondersteund kunnen worden door een netwerk van zorgverleners."

Getuigenis van de zorgverlener:

"Bij ons is iemand daarvoor verantwoordelijk en zij coördineert dat ook. … hier in de stad zijn er verschillende organisaties en zij werken samen, zij werken ook samen met de stad, zij werken dan ook lokaal bij ons, dus er zijn verschillende niveaus…"

Wat bieden we aan?

In dit onderdeel worden handvaten aangeboden om met behulp van een unieke en gestructureerde tool een netwerk te vormen en overleg te houden met zorgverleners uit verschillende praktijken en organisaties.

Enkele begrippen:

- Cirkel: Elk team wordt voorgesteld als een cirkel, die semi-autonoom is. In deze cirkel wordt in plaats van alle macht te centraliseren, deze verspreid onder de verschillende teamleden.
- Rondes: De teamleden communiceren in rondes. Dit betekent dat iedereen aan bod komt en ongeveer evenveel tijd krijgt om te spreken over een topic. Laat de ronde telkens met een ander teamlid beginnen.

Door rondes in te voeren, geef je de teamleden het gevoel dat alle ideeën even belangrijk zijn en zorg je ervoor dat ieder teamlid aandachtig luistert of voorbereid in de vergadering komt.

Werkwijze:

1. Duid een teamlid aan die de cirkel van je organisatie representeert.

2. Het team waarmee uw organisatie wenst samen te werken doet net hetzelfde.

3. Deze twee aangeduide personen zullen telkens de beide meetings volgen en zorgen voor een informatieflow tussen de twee cirkels.

4. Beide personen zijn effectief teamlid in de beide cirkels en kunnen mee beslissingen nemen tijdens overlegmomenten.

5. Het aantal cirkels kan uitgebreid worden afhankelijk van de noden van de teams.

6. Indien nodig kunnen teams voorbereid worden door de tool op blz. 18-19 te volgen.

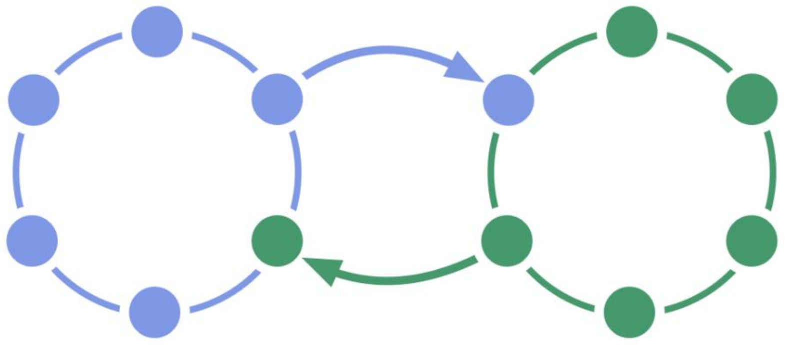

Dubbel-gelinkte cirkels om netwerken te vormen

4.4. Organiseren van speed meetings

Het ontbreekt zorgverleners vaak aan tijd om uitgebreide en geplande meetings te organiseren, waardoor informatiedeling in gedrang kan komen.

Daarnaast weten we dat een aanzienlijk gedeelte van vermijdbare schade bij patiënten wordt veroorzaakt door slechte communicatie. En kunnen snelle, efficiënte en effectieve meetings tussen zorgverleners belangrijke rol spelen om deze schade te voorkomen en om een goede samenwerking, zorgcontinuïteit en zorgcoördinatie te garanderen.

In dit onderdeel reiken we tools aan om snelle en efficiënte overlegmomenten te houden tussen zorgverleners. Deze overlegmomenten duren maximaal 10 minuten en vormen een belangrijke facilitator om te bouwen aan veiligheid in de zorg.

Werkwijze:

Voorafgaand aan een speed-meeting:

1. Gebruik de volgende kanalen om de hulpverlener te bereiken.

- Direct contact
- Kantoor/secretaresse (doordeweeks)
- Privénummer (weekends en na werktijd)
- Mobiel

Voordat de conclusie wordt getrokken dat de hulpverlener niet te bereiken is, moet je al deze opties geprobeerd hebben. Bij spoedsituaties dien je andere bronnen te gebruiken om de veiligheid van de patiënt te verzekeren.

TIP: Als je zijn/haar voorkeur van contactname weet, gebruik die dan. Kondig bijvoorbeeld je eigen beschikbaarheid aan in je mail-signature of briefhoofd. Wacht niet langer dan 5 minuten tussen de verschillende pogingen.

2. Bereid je voor om een efficiënt gesprek met je collega te voeren.

- Heb je de patiënt/cliënt zelf gezien?
- Heb je de casus reeds met andere zorgverleners besproken? Indien ja, wat was hiervan de conclusie?
- Heb je de verslagen van de patiënt/cliënt gelezen?
- Welke zorgverlener is competent om je vraag over de casus op te lossen?
- Houd de volgende documenten bij de hand tijdens het gesprek:
    - Dossier van de patiënt/cliënt
    - Meest recente meting van vitale functies
    - Medicatielijst, allergieën, labo-uitslagen (vermeld hierbij de bron, datum en versie)

3. Tijdens het gesprek met een zorgverlener volg je de SBAR-methode (Situation, Background, Assessment, Recommendation)

Situation/Situatie: Over welk probleem bel je?

- Identificeer de patiënt/cliënt
- Beschrijf het probleem.
- Geef informatie over de ernst van de situatie.
- Geef de data/tijd van de gebeurtenis.

Background/ Achtergrond: Relevante informatie over de achtergrond kan de volgende items bevatten:

- Leeftijd
- Geslacht
- Diagnose
- Medicatieschema
- Datum laatste opname (indien relevant)
- Inhoud laatste consultatie

Assessment/ Beoordeling: Hoe beoordeel je de situatie?

- Meting vitale functies
- Laboresultaten
- Inschatting urgentie
- Beschrijf je conclusies uit je eerste observatie. (Verward, somber, …)
- Opsomming huidige klachten patiënt
- Veranderingen t.o.v. laatste assessment

Reccommendation/Aanbeveling: Wat is jouw aanbeveling, wat wil je dat er gebeurt? Dit kan de volgende items bevatten:

- De patiënt moet langsgaan bij de betreffende zorgverlener
- Dosis of medicatie moet wijzigen. (vb. ten gevolge van een penicilline-allergie)
- Patiënt/cliënt mag een bepaalde test niet ondergaan.

4.5. Hoe evalueer je een team-meeting?

Reserveer als laatste punt van de meeting tijd voor een korte evaluatie. Reflecteer op je interacties met je collega's, geef aan wat er goed of minder goed ging en maak suggesties voor verbetering.

Een reflectie duurt minstens 5 minuten voor elk uur dat je hebt vergaderd. Hieronder zijn er alvast enkele stappen die je kunt ondernemen om deze evaluatie te faciliteren.

Werkwijze:

Evalueer je team-meeting inhoudelijk

Vraag iedereen in een ronde om feedback rond de volgende puntjes:

1. Format van de meeting
2. Participatiegraad en efficiëntie in de meeting
3. Wat apprecieerde je van deze meeting?
4. Welke puntjes zijn er verbeterd sinds de vorige meeting?
5. Welke verbeterpunten zijn er voor de volgende meeting?
6. Zijn er radicale veranderingen nodig in de structuur van de meetings?
7. Extra

5. Gezamenlijke besluitvorming

Zorgverleners die samenwerken, moeten ook samen beslissingen nemen. Dit kan gaan om beslissingen rond de organisatie van de praktijk, of andere praktische zaken, maar ook om patiëntenkwesties.

Bij deze beslissingen gaat het over zorg in samenwerking met alle betrokken partijen, waarbij de patiënt en zijn/haar omgeving centraal staat. Om dergelijke samenwerking te kunnen realiseren, dient een gemeenschappelijke taal te worden gevonden tussen de patiënt en de zorgverlener.

Getuigenis patiënt:

De patiënten drukken hun wens uit om betrokken te zijn in het zorgproces. Ze willen niet behandeld worden als de zoveelste patiënt, maar gezien worden als een persoon. Ze willen samen met de hulpverlener op zoek gaan naar het geschikte zorgtraject. Dit alles draagt bij tot een gevoel van veiligheid en versterkt een vertrouwensband.

Getuigenis zorgverlener:

"Beslissingen worden genomen door consensus. Je moet niet met alles akkoord gaan."

Wat bieden we aan?

In dit onderdeel worden tools aangereikt om efficiënter om te gaan met bedenkingen, om gezamenlijk beslissingen te nemen en akkoorden te documenteren.

Inhoud:

- Hoe ga je om met onenigheden met en tussen je teamleden?
- Hoe los je conflicten of bezwaren van je collega's op?
- Je hebt een akkoord bereikt met je collega's, wat nu?

5.1. Hoe ga je om met bedenkingen van je teamleden?

Tijdens teammeetings worden soms ideeën voorgesteld die mogelijks kunnen botsen met de visie van één of meerdere teamleden. Deze onenigheden worden vaak snel opgelost, maar kunnen in sommige gevallen nefaste gevolgen hebben voor de samenwerking van teams. Hierbij is het belangrijk om na te gaan of de bedenkingen van de teamleden voldoende sterk zijn om te gelden als een bezwaar. In dit onderdeel leer je selectief om te gaan met de bedenkingen van je teamleden.

Getuigenis zorgverlener:

"…maar dan sluit ik mij daar wel bij aan. Er komen ook tegenargumenten en dat is de kracht van een team vind ik."

Werkwijze:

Wanneer iemand een bedenking of tegenargument heeft op je voorstel, luister en probeer deze eerst te begrijpen.

Probeer na te gaan:

- Welke gevolgen zal het wel/niet invoeren van je voorstel hebben op de organisatie?
- Bekijk elk argument of bedenking met aandacht en probeer het goed te begrijpen alvorens een oordeel te hebben.
- Kijk na of de argumenten of bedenkingen kunnen gelden als een volwaardig bezwaar en dat het geen assumpties, persoonlijke voorkeuren of meningen zijn.

Stel je teamleden de vraag:

- Denken jullie dat deze argumenten of bedenkingen sterk genoeg zijn om dit voorstel/akkoord tegen te houden?
- Hoe relateer je de argumenten of bedenkingen met dit specifieke topic of akkoord dat we hebben besproken?
- Kan deze bedenking of argument nu opgelost worden of moeten we hiervoor een nieuw moment inlassen?

5.2. Hoe los je bezwaren van je collega's op?

Je voorstel botste met één of meerdere bedenkingen van je teamleden. De argumenten die ze voorschotelden bleken sterk genoeg om te gelden als een bezwaar. In dit onderdeel leer je als team om te gaan met deze bezwaren.

Getuigenis zorgverlener:

"het is echt de bedoeling dat we als team kijken naar onze frustraties en waar we zelf tegenop botsen want uiteindelijk werk je ook heel sterk met je eigen persoonlijkheid"

Jullie hebben als team besloten dat de bedenkingen van je collega sterk genoeg zijn en kan beschouwd worden als bezwaar. Om dit probleem op te lossen, kan je als team de onderstaande stappen uitvoeren.

1. Pak de bezwaren van je teamleden één voor één aan door ze goed te analyseren en te begrijpen.
2. Begin met het minst ingewikkelde bezwaar, waar je ook het meeste kans hebt op succes.
3. Gebruik het onderstaande schema om de bezwaren op te lossen.

5.3. Je hebt een akkoord bereikt met je collega's, wat nu?

Jullie hebben als team een beslissing genomen. Vergeet dit akkoord zeker niet schriftelijk vast te leggen in een (digitaal) document dat beschikbaar is voor alle betrokken teamleden. In dit onderdeel stellen we jullie een sjabloon voor om jullie afspraken en akkoorden schriftelijk vast te leggen. Dit sjabloon is te vinden op de volgende bladzijde.

Werkwijze:

Dit document moet bevatten:

1. De besproken onderwerpen
2. De gemaakte afspraken
3. Kies een titel per afspraak.
4. Geef de begin- en einddatum van deze afspraak.
5. Datum van de volgende meeting.
6. De rolverdeling en verantwoordelijkheden om de afspraak uit te voeren.
7. Een beschrijving van wat je hebt afgesproken:
    - Wat zijn de verwachtingen?
    - Wat zijn de beperkingen?
    - Wat hebben we nodig om dit te verwezenlijken?
    - Wat willen we bereiken met deze afspraak?

Titel:

Datum/versienummer   :   Beoordelingsdatum:

Wat is de driver van de meeting?

……………………………………………………………………………………………
……………………………………………………………………………………………
……………………………………………………………………………………………
……………………………………………………………………………………………
……………………………………………………………………………………………
……………………………………………………………………………………………
………………………………………………………………………

Wie is verantwoordelijk voor wat? (Naam, verantwoordelijkheid)

- ……………………………………………………………………………………………
- ……………………………………………………………………………………………
- ……………………………………………………………………………………………
- ……………………………………………………………………………………………
- ……………………………………………………………………………………………
- ……………………………………………………………………………………………
  ………………………………………………..

Beschrijving van het akkoord/beslissing

……………………………………………………………………………………………
……………………………………………………………………………………………
……………………………………………………………………………………………
……………………………………………………………………………………………
……………………………………………………………………………………………
……………………………………………………………………………………………
……………………………………………………………………………………………
……………………………………………………………………………………………
……………………………………………………………………………………………

Evaluatiecriteria

……………………………………………………………………………………………….
……………………………………………………………………………………………….
……………………………………………………………………………………………….
……………………………………………………………………………………………….
……………………………………………………………………………………………….

6. Hoe stel je een team (werkgroep) samen rond een specifieke problematiek?

Als zorgverlener ben je betrokken in verschillende processen, binnen en buiten de praktijk. Hoewel deze processen vaak goed verlopen, kunnen er ongetwijfeld ook problemen voorkomen. Uit onze studie blijkt dat veel van deze problemen reeds geïdentificeerd zijn door de zorgverleners, maar niet worden aangepakt. Dit blijken problemen te zijn die niet kunnen opgelost worden als individu, maar waarvoor een team-aanpak nodig is.

Getuigenis zorgverlener:

“Je moet je rol zeker niet zo strikt nemen. Het is niet omdat ik arts ben, dat ik andere problemen niet op mij moet nemen. Dat is soms nodig voor goede zorg.”

Wat bieden wij aan?

In dit gedeelte geven we de zorgverleners een aantal handvaten om deze problemen aan te pakken. We vertrekken vanuit de gedachte: Je hebt een probleem geïdentificeerd als teamlid en wilt een werkgroep opstellen om deze problemen op te lossen. Hoe doe je dit?

Werkwijze:

1. Kijk eerst na of een teamlid reeds bezig is met het oplossen van dat probleem.
    a. Indien ja? Probeer met deze perso(o)n(en) in contact te komen en geef je mening rond het probleem.
    b. Indien neen? Pols bij collega's wat hun visie is rond deze problematiek.
    c. Kijk naar de haalbaarheid, kosten/baten-ratio,… en neem een beslissing na overleg met je collega's over het al dan niet vormen van een werkgroep.

2. Kijk na wie er betrokken is in dit probleem en wie er wordt beïnvloed door dit probleem
   Zo heb je meer kans op gemotiveerde collega's tijdens het werken aan specifieke problematiek.

3. Maak voor jezelf een korte lijst van collega's die mogelijks kunnen participeren:
    a. Wie heeft interesse in het oplossen van dit probleem?
    b. Wie wordt er beïnvloed door dit probleem?
    c. Wie bezit de juiste profiel/competenties om dit probleem op te lossen?
    d. Wie heeft er baat bij als dit probleem wordt opgelost?
    e. Welke collega's kunnen hier tijd voor maken?

   Tip: Maak een lijst op met de personen die volgens jou voldoen aan deze criteria en start met het contacteren van deze personen. Afhankelijk van de proportie van of het type probleem zal deze lijst met personen variëren. Zie deel 2 als je twijfels hebt over hoe je je collega's het best kunt contacteren.

4. Motiveer je collega's om te participeren door uit te leggen wat dit hen kan opleveren. Hieronder vind je alvast enkele suggesties:
    a. Een goed gevoel omdat ze de zorg voor de patiënt/cliënt kunnen verbeteren.
    b. Krijgen van erkenning en respect.
    c. Beter leren kennen van je collega's.
    d. Nieuwe ervaring opdoen en nieuwe competenties aanleren.

5. Wat verwachten je collega's van jou en welke bijdrage kan je van hen verwachten?

    a. Maak eerst duidelijk welke bijdrage ze van jou mogen verwachten.
    b. Maak duidelijk welke bijdrage je van hen verwacht.
    c. Kijk na of deze overeenkomen met de visie en doelen van de praktijk en de opdracht.
    d. Maak samen met je team een planning waarin je rekening houdt met de beschikbaarheid van de teamleden.

7. Hoe werk je persoonsgericht?

Wat is persoonsgerichte zorg:

Persoonsgerichte zorg is het behandelen van een persoon/patiënt, op een eervolle en respectvolle manier. Waarbij de patiënt wordt betrokken in alle beslissingen die worden genomen in de zorg die hij/zij krijgt.

Door meer persoonsgericht te werken kan de zorgverlener, de patiënt en zijn/haar omgeving beter van dienst zijn. De patiënt verwacht dat de zorgverlener hem/haar ziet als een partner in de zorg en hoopt dat er rekening wordt gehouden met zijn voorkeur, wensen en doelen. Ze willen meebeslissen in de zorg en behandeling die ze krijgen.

Hierdoor is de patiënt beter geïnformeerd en zien we een verhoogde therapietrouw en een betere relatie tussen zorgverlener en patiënt.

Getuigenis zorgverlener:

“Het is wel de bedoeling dat we … kijken, wat zijn de noden van onze patiënt, wat zijn de noden van de wijk en wat sluit daarbij aan. Maar dat gaat dan … niet zozeer over echt medisch inhoudelijk of gezondheid.”

“Om ervoor te zorgen dat we zeker aansluiten op wat dat patiënten nodig hebben, willen we dat ook expliciet bevragen”

Werkwijze voor persoonsgerichte zorg:

- Voorzie je patiënt van een waardige, compassie- en respectvolle behandeling.
- Biedt een goed gecoördineerde en geïntegreerde zorg, begeleiding en behandeling.
- Biedt een gepersonaliseerde zorg, begeleiding en behandeling aan.
- Ondersteun en begeleid je patiënten zodat ze zichzelf kunnen verbeteren en opdat ze meer zelfstandig kunnen leven.
- Geef je patiënten emotionele steun
- Maak zorg laagdrempelig en toegankelijk
- Houdt rekening met de familie en omgeving van de patiënt.
- Geef je patiënt zekerheid omtrent continuïteit van zorg.

Wat bieden we aan?

In dit onderdeel geven we tools aangereikt om samen te werken met de patiënt en zijn/haar familie en omgeving.

8. Hoe integreer je een nieuw teamlid?

Je hebt vernomen dat je een nieuwe collega krijgt en wilt ongetwijfeld zo snel mogelijk kennis maken. Hoewel dit goed nieuws en vele opportuniteiten met zich meebrengt, is er ook heel wat werk aan verbonden. Daarnaast is een vlotte, correcte integratie van een nieuw teamlid cruciaal voor het welzijn van jou, je collega's en de werking van de praktijk.

Ervaring zorgverlener:

"We hebben ook wel een manager ingeschakeld die ons geholpen heeft om functieprofielen uit te schrijven, taakomschrijvingen en dan te kijken wie er nodig is of op welke manier we best een invulling van een bepaalde taak kunnen organiseren."

Wat bieden we aan?

In dit onderdeel bieden we tools aan om jou en je collega's te begeleiden in het integreren van een nieuw teamlid.

Stap 1: Nog voor jullie aanwinst arriveert kan je je huidige team reeds voorbereiden op de komst van je nieuwe collega. Wees hierbij zeker niet te voorzichtig en verspreid het goede nieuws aan al je teamleden.

Stap 2: Verwelkom je collega en introduceer eerst jezelf. Dit lijkt een simpele actie, maar is zeer effectief in het faciliteren van het integratieproces van een nieuw teamlid. Probeer reeds kennis te maken en zoek naar overeenkomsten zoals: hobby's, …

Stap 3: Neem initiatief en stel hem/haar voor aan je collega's. Jouw insteek maakt deze kennismaking vele malen gemakkelijker.

Stap 4: Maak duidelijk wat de visie, waarden, doelen en prioriteiten van jullie team zijn. Wat vinden jullie belangrijk? Wat staat voorop? Geef hierbij telkens aan dat je openstaat voor vragen.

Stap 5: Leg aan je nieuwe collega uit hoe de rollen en daaraan gelinkte verantwoordelijkheden zijn verdeeld. Begin hierbij eerst met de individuele rollen en verantwoordelijkheden en ga dan over (indien van toepassing) naar de gemeenschappelijke.

Stap 6: Maak gebruik van de lunchpauzes om je collega beter te leren kennen en zorg ervoor dat hij/zij vertrouwd raakt met de omgeving.

Stap 7: Maak duidelijk dat je nieuwe collega met al zijn/haar vragen bij jou of je andere collega's terecht kan. Dit kan vanzelfsprekend klinken voor jou, maar is niet altijd gemakkelijk voor een nieuw teamlid.

Bijlagen:

A. Meer informatie over de gebruikte concepten

Dit onderdeel wordt aangevuld op basis van onduidelijkheden die de gebruikers melden.

Over interprofessionele samenwerking

Interprofessionele samenwerking is wanneer twee of meer professies samenwerken om een gemeenschappelijke doelstelling te bereiken. Dit concept wordt vaak gebruikt om verschillende complexe problemen op te lossen.

Deze vorm van samenwerking ontstaat volgens de WHO wanneer meerdere zorgverleners met verschillende professionele achtergronden samenwerken met patiënten, families, verzorgers en de gemeenschap om de hoogste kwaliteit van zorg te leveren.

Over psychologische veiligheid

Er is sprake van psychologische veiligheid wanneer mensen het gevoel hebben zich te kunnen uiten ten aanzien van anderen, zonder angst voor consequenties en negatieve kritiek. Het gaat om het vervangen van het gevoel van angst voor kritiek, door het gevoel van respect en toegankelijkheid.

Het wordt door Edmondson voorgesteld als een voorwaarde om tot goede samenwerking te komen en is een strategie voor *integratie* of hoe maximaal gebruik te maken van ieder teamlid zijn/haar competenties. Als een psychologisch veilige omgeving verzekerd is, worden teamleden aangemoedigd om hun ideeën en gedachten zonder gevoel van angst te uiten. Daarnaast zorgt een psychologisch veilige omgeving ervoor dat teamleden beter presteren in hun individuele taken, meer innovatief kunnen denken en meer voldoening halen uit hun werk.

Over sociocratie 3.0

Sociocratie 3.0 (S3) is een databank waar materiaal gratis beschikbaar wordt gesteld. De oprichting ervan gebeurde in maart 2015 door Bernhard Bockelbrink en James Priest en iets later door het team van Liliana David. Ze hebben alle drie samen een uitgebreide ervaring in communicatie, management, bedrijfskunde, agility en lean, softwareontwikkeling en organisatie coaching, zowel in binnen- als buitenland. S3 baseert zich op de sociocratische bestuursvorm, maar wordt nog verder ontwikkeld. Methoden zoals de Sociocratische Kring Organisatiemethodiek, Lean-management, Kanban en andere technieken worden geïmplementeerd. Het bevat veel elementen uit agile werkwijzen. Het Engelse woord *agile* betekent behendig, lenig. Agile technieken, zoals Lean Management, kennen hun oorsprong in de ontwikkeling van software, maar worden tegenwoordig meer en meer gebruikt in de gezondheidszorg

S3 is gebaseerd op de sociocratie, een bestuursvorm die uitgaat van de gelijkwaardigheid van individuen. Besluiten worden hier genomen aan de hand van ‘consent’. Dit betekent dat een besluit kan genomen worden als er geen bezwaren zijn bij de teamleden tegen het nemen van dat besluit. Indien er wel beargumenteerde bezwaren zijn, wordt het voorstel aangepast tot de bezwaren verholpen zijn. ‘Consent’ voorkomt zo de valkuil van de consensus, waar iedereen akkoord moet gaan met een besluit. En in tegenstelling tot een democratische bestuursvorm waar het idee van de minderheid kan worden onderdrukt, worden bij sociocratie alle ideeën mee in overweging genomen.

De zeven principes van sociocratie:

1. Transparantie: Maak alle informatie beschikbaar voor iedereen in de organisatie, tenzij er een reden is voor vertrouwelijkheid
2. Gelijkwaardigheid: Betrek mensen bij het maken en evalueren van afspraken die hen raken
3. Consent: Geef, zoek en integreer bezwaren tegen beslissingen en acties
4. Continu verbeteren: Breng continu kleine veranderingen aan om empirisch leren mogelijk te maken
5. Accountability: Reageer wanneer iets nodig is, doe waar je mee hebt ingestemd en neem verantwoordelijkheid voor de koers van de organisatie
6. Empirisme: Test alle veronderstellingen door voortdurend te experimenteren en evalueren
7. Effectiviteit: Investeer alleen tijd in die dingen die je dichter brengen bij het bereiken van je doelen

Over het SBAR-model

Met de SBAR-methode (Situation, Background, Assessment, Recommendation) is het mogelijk om de communicatie en coördinatie rond en over een patiënt tussen verschillende hulpverleners (bijvoorbeeld tussen de arts en de verpleegkundige) te verbeteren. Door de verschillende aspecten (…) te bespreken, is er zeer snelle (<5 min) en efficiënte communicatie mogelijk, waarna zorgverleners terug aan het werk kunnen. Deze methode kan zowel bij fysieke als bij telefonische meetings gebruikt worden.

Hoewel dit model vaak wordt toegepast in ziekenhuizen, wordt het alsmaar meer geïntegreerd in eerstelijnspraktijken.

Over het model voor gezamenlijke besluitvorming

Shared decision making of gezamenlijke besluitvorming is het gezamenlijk komen tot een beslissing door een hulpverlener (of andere professional) en een patiënt. Een bekende auteur en expert op het gebied van Shared decision making is Glyn Elwyn, hij definieert het als het proces waarin de zorgverlener en de patiënt samen beslissingen nemen over diagnostiek, behandelingen of begeleiding, op grond van (wetenschappelijke) kennis, klinische ervaring en de voorkeuren en waarden van de patiënt (Elwyn 2012).

B. Woordenlijst

Deze woordenlijst wordt aangevuld op basis van onduidelijkheden die de gebruikers melden.

Afgevaardigde: Zorgt ervoor dat info wordt gedeeld met zorgverleners buiten de cirkel.

Backlog: Oplijsting (o.b.v. prioriteiten) en planning van onafgewerkte taken die nog moeten aangepakt worden.

Cirkel: Elk team wordt voorgesteld als een cirkel, die semi-autonoom is. In deze cirkel wordt alle macht verspreid onder de verschillende teamleden, i.p.v. ze te centraliseren bij één iemand.

Facilitator: Zorgt ervoor dat meetings vlot verlopen en dat iedereen in de meeting zeggenschap heeft.

Leider/coördinator: Zorgt ervoor dat alles volgens de visie van de organisatie verloopt en dat de einddoelen worden behaald.

Rondes: De teamleden communiceren in rondes. Dit betekent dat iedereen aan bod komt en ongeveer evenveel tijd krijgt om te spreken over een topic. Laat de rondes telkens door een ander teamlid beginnen.

Secretaris: Teamlid dat alle documenten onderhoudt die te maken hebben met overlegmomenten.

Semi-autonoom: Elk teamlid of team handelt semi-autonoom. Dit betekent dat een teamlid handelt binnen de beperking van zijn/haar eigen domein of dat elk team autonoom eigen waarden en afspraken kan ontwikkelen.

Tolerantie bereik: Tussen de voorkeur en het onaanvaardbare ligt het tolerantiebereik van mensen. Door te werken binnen dit tolerantiebereik, kan een team de zoektocht naar flexibiliteit en perfectie optimaliseren.

Referenties

Kringos, D.S., et al., *Building primary care in a changing Europe*. 2015: World Health 1003 Organization. Regional Office for Europe.

Pellegrin-Boucher, E., F. Le Roy, and C. Gurău, *Coopetitive strategies in the ICT sector:* 1023 *typology and stability.* Technology Analysis & Strategic Management, 2013. **25**(1): p. 71-89. 1024

Srivastava, A., S. Bhardwaj, and S. Saraswat. *SCRUM model for agile methodology*. in *2017* 1025 *International Conference on Computing, Communication and Automation (ICCCA)*. 2017. 1026 IEEE.

re:Work. Guide: Understand team effectiveness. 2017 21/04/2022]; Available from: 1040 https://rework.withgoogle.com/print/guides/5721312655835136/.

Newman, A., R. Donohue, and N. Eva, Psychological safety: A systematic review of the 1042 literature. Human resource management review, 2017. 27(3): p. 521-535. 1043

Edmondson, A., Psychological Safety and Learning Behavior in Work Teams. Administrative 1044 Science Quarterly, 1999. 44(2): p. 350-383. 1045

Edmondson, A.C., The fearless organization: Creating psychological safety in the workplace 1046 for learning, innovation, and growth. 2018: John Wiley & Sons.

Eckstein, J. Sociocracy: An organization model for large-scale agile development. in 1052 Proceedings of the Scientific Workshop Proceedings of XP2016. 2016. 1053

Ward, L.F., Sociocracy, in The psychic factors of civilization. 1892, Ginn & Company: 1054 Boston, MA, US. p. 313-331. 1055

Eckstein, J. and J. Buck, Company-wide agility with beyond budgeting, open space & 1056 sociocracy. Survive & thrive on disruption. Kindle cloud edition, 2018. 23: p. 2018. 1057

Owen, R.L. and J.A. Buck, Creating the conditions for reflective team practices: examining 1058 sociocracy as a self-organizing governance model that promotes transformative learning. 1059 Reflective Practice, 2020. 21(6): p. 786-802. 1060

Bockelbrink, B., J. Priest, and L. David, A Practical Guide for Evolving Agile and Resilient 1061 Organizations with Sociocracy 3.0. 2017: USA. 1062

Christian, D.L., Transparency, equivalence, and effectiveness: How sociocracy can help 1063 communities, part I. Communities, 2013(160): p. 59.

Christian, D.L., Self-governance with circles and double links: How sociocracy can help 1065 communities, Part II. Communities, 2013(161): p. 61. 1066

Christian, D.L., Why no tyranny of the minority in sociocracy: How sociocracy can help 1067 communities, Part IV. Communities, 2014(165): p. 60.

Sirimsi, M.M., et al., Scoping review to identify strategies and interventions improving 1086 interprofessional collaboration and integration in primary care. BMJ Open, 2022. 12(10): p. 1087 e062111.

Kostoff, M., et al., An Interprofessional Simulation Using the SBAR Communication Tool. 1019 American Journal of Pharmaceutical Education, 2016. 80(9): p. 157. 1020

Müller, M., et al., Impact of the communication and patient hand-off tool SBAR on patient 1021 safety: a systematic review. BMJ open, 2018. 8(8): p. e022202.

Cumps, J. (2019). Sociocracy 3.0-The Novel: Unleash the Full Potential of People and Organizations. Lannoo Meulenhoff-Belgium.
