# Supplementary material for: Development of a toolkit to improve interprofessional collaboration and integration in primary care using qualitative interviews and co-design workshops
Source: Front Public Health. 2023 Apr 17;11:1140987. doi: 10.3389/fpubh.2023.1140987 (PMC10149845; doi:10.3389/fpubh.2023.1140987)

Supplementary Material

Development of a toolkit to improve interprofessional collaboration and integration in primary care using qualitative interviews and co-design workshops

**Muhammed Mustafa Sirimsi^1,2*^, Hans De Loof^3^, Kris Van den Broeck^1^, Kristel De Vliegher^4^, Paul Van Royen^1^, Peter Pype^5^, Kristel Driessens^6^, Emily Verté^1,7^, Roy Remmen^1^, Peter Van Bogaert^2^**

*** Correspondence:** Muhammed Mustafa Sirimsi: Muhammedmustafa.sirimsi@uantwerpen.be

# Supplementary Data: Interview guides

**1.1. Interview guide 1**

Icebreaker: I suggest we do a round where everyone can briefly introduce themselves, stating your name, work setting and relevant work experience.

Question 1: How do you experience communication between caregivers within your team?

- Good and bad examples?
- Who do you (still) come into contact with?
- What tools are used? Role of digitalisation?
- How do you communicate with carers outside your team?

Question 2: Do you organise team meetings?

- If yes:

- How are these conducted? (good/bad example)
- What makes your meetings good or bad?
- How often do you meet?
- What methods do you use?

- If no:

- What is the reason?
- Is there an alternative you use? And what?

Question 3: Are there any digital meetings (can also be answered in question 2)?

- If yes:

- How are these conducted? (good/bad vb)
- What makes your meetings good or bad?
- How often do you meet digitally?
- What meeting methods do you use?

- If no:

- What is the reason?
- Is there an alternative you use? And what?

Question 4: How is information sharing within your team?

- With other practices or organisations

- Role of technology/digitisation

Question 5: How do you see the relationship between interprofessional collaboration and quality of care?

- Positive and negative examples.

- How can collaboration improve quality in care?

Question 6: What implementations or interventions have led to improvements in your collaboration?

- Interventions on information sharing, communication, coordination,...

- What makes your collaboration good or bad?

**1.2. Interview guide 2**

**Opening**

Would you like to introduce yourself briefly? What is your position within your organisation and in what way did you become involved in this collaborative initiative?

**Establishment of your organisation**

How did you come up with the idea of working together in this way? From where is this initiative being organised?

Which objectives are already being achieved at the moment? Which objectives would you like to achieve in the future?

- Are you getting enough resources from the government?

**Functioning of the organisation**

Is there a collaboration between primary care and secondary care? How does this collaboration work?

- How do you experience interprofessional collaboration within your organisation
- How does your organisation adapt to (demographic) changes and evolutions in the neighbourhoods/environment/population?

How does this collaboration work in concrete terms?

In what way are decisions made?

How do team meetings take place? What does such a meeting look like?

What does ideal care for clients look like for you?

What is needed to obtain this ideal care?

What makes it difficult? What are the weaknesses of this interprofessional collaboration? What is currently not going well and needs to change to improve care for clients?

What are favourable factors? What are the strengths of this interprofessional collaboration? What is already going well and would you like to maintain?

**Round-up**

Which three factors could improve collaboration in the future?

**1.3. Interview guide 3**

Icebreaker: I suggest we do a round where everyone can briefly introduce themselves, stating your name, position/role within your work setting and work experience.

1. How is your teamwork structured?

- Who takes on which roles/tasks (go deeper into role division)

- How is this agreed upon? How are your roles defined/adjusted?
- What about team arrangements in general? How does that function?

- Looking at the beginning of your teamwork and now, how has your teamwork evolved in that time span?

- How did you arrive at the current team functioning?
- How does a change in the composition of your team happen?

2. From what shared objective did you make the decision to work together as a team?

- Does your team have a "mission statement"?

- Is this objective/mission explicit or implicit?
- Is this something changeable? Is there e.g. a fixed moment to reflect on it, or a meeting to review it?

- To what extent do you achieve this objective?

- What contributes to this?
- What makes it difficult?

Ask some questions on care substitution, working groups (how established, autonomy?)

And also executive board and board of directors, policy plan,…

3. How is the collaboration within your team?

- In what way do you communicate? Tools?

- What goes well - less well - why?
- Electronic file, telephone,...

- How do you exchange data (role of technology)?

- (digital) consultation moments?
- What do such consultation moments entail and how are they conducted?

- What needs to change/is needed to improve communication within your team?

If we look at this a little more broadly and include caregivers outside your regular team.

4. How is your collaboration with caregivers outside your team going?

- What goes well - What goes less well - Why?

- How do you exchange data (role of technology)?

- - (digital) consultation moments?
  - What do these consultation moments entail and how do they take place?

- What do you need to improve communication with them?

And if we extend this to the patient.

5. How do you coordinate care around the patient and to what extent is the patient involved in this?

- What goes well - What goes less well - Why?

- How is the patient involved in this coordination?

- What do you need to improve coordination around and with the patient?

6. What effect does working as an interprofessional team have on the quality/quantity of care?

- Positive and negative examples.

- What makes this form of collaboration improve the quality of care?

7. What introductions or interventions have led to improvements in your collaboration? (if not listed in the previous questions)

- When you hire a new team member, how is he or she incorporated into the group?

- Interventions on information sharing, communication, coordination,...

Round-up

Give me 1 element needed to improve the communication and coordination of your practice.

# Supplementary Figures

**Supplementary Figure 1.** An overview of the two-year development process of the IPCI-toolkit .
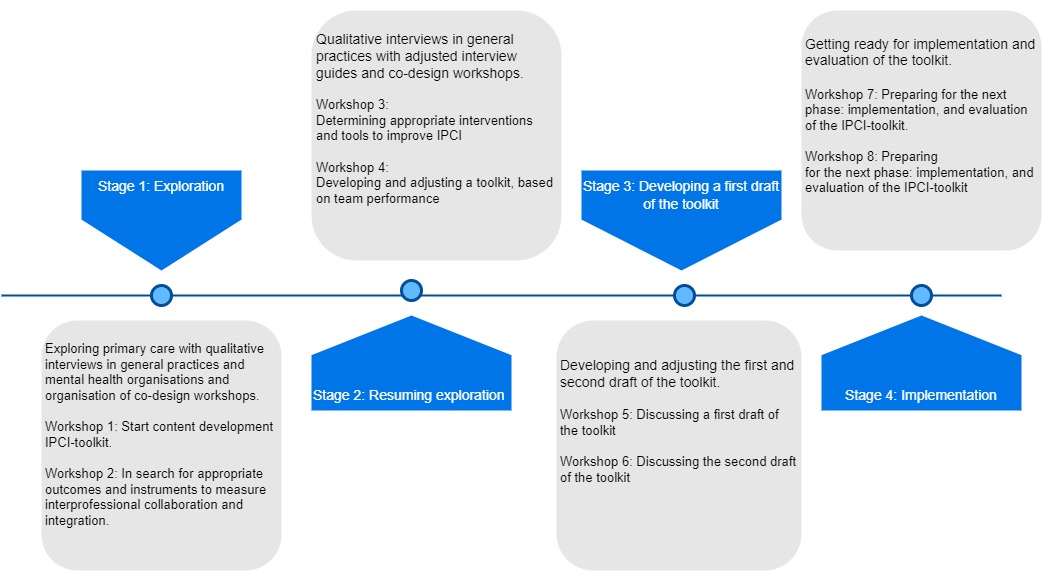

Supplement: Supplementary file 3 [file Table_1.DOCX]
